# Genome and pan-genome assembly of asparagus bean (*Vigna unguiculata* ssp. *sesquipedialis*) reveal the genetic basis of cold adaptation

**DOI:** 10.3389/fpls.2022.1059804

**Published:** 2022-12-16

**Authors:** Le Liang, Jianwei Zhang, Jiachang Xiao, Xiaomei Li, Yongdong Xie, Huaqiang Tan, Xueping Song, Li Zhu, Xinru Xue, Linyu Xu, Peihan Zhou, Jianzhao Ran, Bo Sun, Zhi Huang, Yi Tang, Lijin Lin, Guochao Sun, Yunsun Lai, Huanxiu Li

**Affiliations:** ^1^ College of Horticulture, Sichuan Agricultural University, Chengdu, Sichuan, China; ^2^ Vegetable Germplasm Innovation and Variety Improvement Key Laboratory of Sichuan, Chengdu, China; ^3^ Institute for Processing and Storage of Agricultural Products, Chengdu Academy of Agriculture and Forestry Sciences, Chengdu, China; ^4^ Horticulture Research Institute, Chengdu Academy of Agriculture and Forestry Sciences, Chengdu, China; ^5^ Institute of Pomology and Olericulture, Sichuan Agricultural University, Chengdu, Sichuan, China

**Keywords:** *De novo* assembly, evolution, pan-genome, presence/absence variation, *Vigna unguiculata* ssp. *sesquipedialis*

## Abstract

Asparagus bean (*Vigna unguiculata* ssp. *sesquipedialis*) is an important cowpea subspecies. We assembled the genomes of Ningjiang 3 (NJ, 550.31 Mb) and Dubai bean (DB, 564.12 Mb) for comparative genomics analysis. The whole-genome duplication events of DB and NJ occurred at 64.55 and 64.81 Mya, respectively, while the divergence between soybean and *Vigna* occurred in the Paleogene period. NJ genes underwent positive selection and amplification in response to temperature and abiotic stress. In species-specific gene families, NJ is mainly enriched in response to abiotic stress, while DB is primarily enriched in respiration and photosynthesis. We established the pan-genomes of four accessions (NJ, DB, IT97K-499-35 and Xiabao II) and identified 20,336 (70.5%) core genes present in all the accessions, 6,507 (55.56%) variable genes in two individuals, and 2,004 (6.95%) unique genes. The final pan genome is 616.35 Mb, and the core genome is 399.78 Mb. The variable genes are manifested mainly in stress response functions, ABC transporters, seed storage, and dormancy control. In the pan-genome sequence variation analysis, genes affected by presence/absence variants were enriched in biological processes associated with defense responses, immune system processes, signal transduction, and agronomic traits. The results of the present study provide genetic data that could facilitate efficient asparagus bean genetic improvement, especially in producing cold-adapted asparagus bean.

## 1 Introduction

Cowpea (*Vigna unguiculata*), a diploid (2n = 22) in the family Fabaceae, is a staple food crop globally. The plant was originally domesticated in sub-Saharan Africa and is closely related to several protein-rich warm-season beans (Lonardi et al., 2019). Asparagus bean (*Vigna unguiculata* ssp. *sesquipedialis*) is a cowpea subspecies mainly grown for its 50−100-cm tender green pods. The pods are high in protein, organic acids, polyols, monosaccharides, and fatty acids.

Asparagus bean is subject to various abiotic stresses, including heat and drought (Xu et al., 2015), salt (Pan et al., 2019), and cold stresses (Heidarvand et al., 2017), affecting primary metabolism, respiration rate, and energy supply for growth. Cold stress is a major limiting factor affecting plant distribution and growth and can significantly alter plant cell characteristics (Heidarvand et al., 2017). Asparagus bean planted in early spring is often challenged by cold stress, which greatly affects subsequent yield and quality. Huang et al. (2018) performed high-throughput specific-locus amplified fragment sequencing of Ningjiang 3 (hereafter NJ) and Dubai bean (hereafter DB), and 5,225 single nucleotide polymorphism (SNP) markers were developed to construct a genetic map (consisting of 11 linkage groups) with a total length of 1,850.81 cM with an average distance of 0.35 cM between markers. In that study, NJ seedlings exhibited better cold tolerance than DB seedlings. Interestingly, the cold tolerance of these two cultivars at the pod setting stage was opposite to that at the seedling stage (Tan et al., 2016). Comparative transcriptome analysis of NJ and DB seedlings under 5 °C cold stress and 25 °C recovery conditions revealed that the differentially expressed genes were enriched mainly in the Gene Ontology (GO) terms for protein phosphorylation, photosynthesis, and redox processes (Miao et al., 2022).

A 519-Mb haploid genome of IT97K-499-35, with 722 scaffolds and 11 chromosomes, has previously been assembled, a phylogenetic tree constructed, and the genomes of some superfamilies analyzed (Lonardi et al., 2019). Additionally, Xia et al. (2019) assembled a 632.8-Mb genome (549.81 Mb non-N size) of the asparagus bean (Xiabao II) based on whole-genome shotgun sequencing and generating a linkage map. The genome assembly has facilitated greater insights into the diversity of cowpea species at the SNP level (Della Coletta et al., 2021). With the development of various sequencing technologies, a wider range of structural variants (SVs), presence/absence variants (PAVs), and copy number variations (CNVs) have been discovered (Sun et al., 2022). The SVs affect the gene density and gene copy number of the genome, highlighting the limitations of a single genome (Li et al., 2014; Willson, 2020; Della Coletta et al., 2021). Pan-genome analysis is mining re-sequenced individual variant genes, including core and variable genes, where variable genes may produce different traits (biotic or abiotic resistance, flowering, and cold resistance) (Razzaq et al., 2021). PAVs are one of the focus points in pan-genome research (Zanini et al., 2022). Recently, pan-genomics research has been carried out in rice (Qin et al., 2021), maize (Springer et al., 2018), cotton (Li et al., 2021), eggplant (Barchi et al., 2021), cucumber (Li et al., 2022), strawberry (Qiao et al., 2021), and legumes, i.e., soybean (Torkamaneh et al., 2021; Ruperao et al., 2021), barrel medic (Zhou et al., 2017), and pigeon pea (Zhao et al., 2020). Sequencing and *de novo* assembly of seven geographically representative accessions in the soybean pan-genome identified lineage-specific genes and genes with large effect mutations. Some showed positive selection and may lead to variation in various agronomic traits (Li et al., 2014). Based on 15 *de novo* assemblies of barrel medi, the dispensable genes had greater variability than the core genes. Additionally, 22% of the genome was involved in large structural changes, expanding the reference genome by 16% (630 Mb). Rapidly evolving gene families are mainly enriched in regions related to biological interactions and stress responses (Zhou et al., 2017). Zhao et al. (2020) constructed the first pigeon pea pan-genome in which the annotation of variable genes suggested that they were associated with self-fertilization and response to disease and identified 225 SNPs associated with nine important agronomic traits. Although the genome of the asparagus bean has been assembled, there is no report on its origin and pan-genome.

In this study, the genomes of NJ and DB were assembled and compared with the genomes of 11 other plants to analyze the evolution and origin of the asparagus bean. A pan-genome of NJ, DB, IT97K-499-35 and Xiabao II was constructed using NJ as a reference genome. Small (SNPs and InDels) and large (SVs, CNVs, and PAVs) variants in the genome were mined, a high-density genetic map was reconstructed. This information will provide a theoretical basis for the molecular mechanism of intraspecific variation and cold tolerance of *Vigna* and further research on gene regulation.

## 2 Materials and methods

### 2.1 Genome sequencing, assembly, and evaluation

Two varieties of asparagus bean, Ningjiang 3 (NJ) and Dubai bean (DB), were used for genome sequencing. High-quality genomic DNA was extracted from the leaves of three-week-old plants using a modified CTAB method. The extracted DNA was sequenced with Illumina Novaseq 6000 (Illumina Inc., San Diego, CA, USA) and PacBio Sequel II (Pacific Biosciences of California, Menlo Park, CA, USA) platforms. The long reads from the PacBio platform were used for genome assembly. The short reads from the Illumina platform were quality filtered using HTQC v1.92.310 and used to estimate the genome size, level of heterozygosity, and repeat content. The assembled genome was assessed and Cegma v2.5 was used to evaluate the integrity of the final genome assembly by aligning short reads from the Illumina platform to IT97K-499-35 (Lonardi et al., 2019). The uniquely mapped data were retained to perform assembly by using LACHESIS software.

### 2.2 Transposable elements and tandem repeat annotations

TEs were identified by a combination of homology-based and *de novo* approaches. We customized a *de novo* repeat library of the genome using repeatModeler, automatically executing RECON v1.08 and repeat Scout to find two *de novo* repeats. Subsequently, the high-quality intact full-length long terminal repeat retrotransposons (fl-LTR-RTs) and non-redundant LTR library were identified using both LTRharvest, LTR_finder, and LTR_retriever. A non-redundant species-specific TE library was constructed by combining the *de novo* TE sequences library above with the known Repbase v19.06, REXdb v3.0, and Dfam v3.2 databases. Final TE sequences were identified and classified by homology search against the library using RepeatMasker v4.10. Tandem repeats were annotated by Tandem Repeats Finder and MIcroSAtellite identification tool MISA v2.1.

### 2.3 Gene prediction and annotation

We integrated three approaches, namely, *de novo* prediction (Augustus v2.4 and SNAP); homology search (GeMoMa v1.7); and transcript-based assembly (RNA-sequencing data were mapped to the reference genome (IT97K-499-35) using Hisat v2.0.4 and assembled by Stringtie v1.2.3) to annotate protein-coding genes in the genome. GeneMarkS-T v5.1 was used to predict genes based on the assembled transcripts. Gene models from the different approaches were combined using EVM v1.1.1 (Haas et al., 2008) and updated using PASA. The final gene models were annotated by searching the GenBank Non-Redundant (NR, 20200921), TrEMBL (202005), Pfam (v33.1), SwissProt (202005), GO (20200615), and KEGG (20191220) databases.

### 2.4 Annotation of non-coding RNA and pseudogenes

We used tRNAscan-SE v2.0.9 algorithms and Barrnap v0.9, both with default parameters, to identify the genes associated with tRNA and rRNA, respectively. MiRNAs and snRNAs were identified using Infernal v1.1.1 software against the Rfam v14.5 database with default parameters. After masking predicted functional genes, the GenBlastA v1.0.4 program was used to scan the whole genomes. Putative candidates were then analyzed by searching for non-mature and frame-shift mutations using GeneWise v2.4.1. References and software data are supplied as supplementary information.

### 2.5 Gene family identification

We used proteins from the longest transcripts of each gene from NJ, DB, and *Oryza sativa*, *Spinacia oleracea*, *Solanum tuberosum*, *Raphanus sativus*, *Lupinus albus*, *Cajanus cajan*, *Medicago truncatula*, *Glycine max*, *Vigna angularis*, *V. radiata*, and *V. unguiculata* to cluster the protein-coding gene families. The protein-coding sequences were compared using OrthoFinder v2.4.0. The PANTHER v15 database was used to annotate the gene families and perform GO and KEGG enrichment analyses for the species-specific gene family.

### 2.6 Phylogenetic analysis

The protein sequences of the 469 single-copy orthologous genes were aligned using the MAFFT v7.205 program. The corresponding CDS alignments were generated and concatenated using protein alignment. IQ-TREE v1.6.11 was used to construct the phylogenetic tree. Using *O. sativa* as the outgroup, the divergence time was calculated using PAML v4.9i (supplied with MCMCTREE). The final evolutionary tree with differentiation time was obtained using MCMCTreeR v1.1. Geological periods used were Permian (Pe), Triassic (Tr), Jurassic (Ju), Cretaceous (Cr), Paleogene (Pa), Neogene (Ne), and Quarternary (Qu). References and software data are supplied as supplementary information. From TimeTree (http://www.timetree.org/), we obtained a species evolutionary tree with divergence time.

### 2.7 Gene family expansion and contraction analysis

We used CAFÉ to analyze the expansion and contraction of gene families. The criteria for significant expansion or contraction of a gene family are that both family-wide P-values and viterbi *P*-values are < 0.05. GO and KEGG enrichment analyses were performed on the expansion and contraction gene families of NJ and DB. References and software data are supplied as supplementary information. Positively selected genes and whole genome duplication analysis.

We mainly used the CodeML module in PAML for positive selection analysis. Firstly, to obtain single-copy gene families among *V. angularis*, *V. radiata*, *V. unguiculata*, DB, and NJ, we used MAFFT (parameter: –localpair –maxiterate 1000) to compare the protein sequences of each gene family. Subsequently, we reverted to codon-aligned sequences using PAL2NAL. Finally, we used CodeML based on the Branch-site model to pair the two models, and the significant differences were evaluated (*P* < 0.05). The Bayesian method was used to obtain the posterior probability of a site considered positively selected (usually > 0.95 indicates a significantly positively selected site). GO and KEGG enrichment analyses for positively selected genes were then performed. We used WGD v1.1.0 and a custom script to identify WGD events.

### 2.8 Pan-genome construction and gene family analysis

Four accessions (NJ-the reference genome, DB, IT97K-499-35 and Xiabao II) were used for pan-genome construction based on *de novo* alignment. The genome sequences of the NJ and DB parents were aligned with the reference genome sequences using MUMmer v4.0, and the pan-genome was constructed using ppsPCP. The gene families were defined as follows: (i) Core gene, gene family shared by the four species, (ii) Dispensable gene, gene family shared by two or three species, and (iii) Unique gene, the specific gene family for each species. The protein sequences of the three species were classified using OrthoFinder v2.3.7 software. The distribution map of the presence and absence of gene families was constructed according to the gene family clustering results. The GO and KEGG enrichment analysis was performed for the core, dispensable, and unique gene families. References and software data are supplied as Supplementary material.

### 2.9 Sequence variation analysis

Whole-genome alignment was performed using MUMmer 4.0. Synteny and Rearrangement Identifier (SyRI) detects variants using default parameters and identifies collinearity regions, structural rearrangements (inversions, translocations, and duplications), local variations (SNPs, InDels, SVs, PAVs, CNVs), and regions not aligned. A variation with a sequence length > 50 bp that does not exist in the NJ is defined as “presence”. Variation that exists only in the NJ is defined as “absence”. After detection, SNPs and InDels were annotated using the ANNOVAR software toolkit. We performed GO and KEGG enrichment analysis for the variable genes. References, software data, and other content are supplied as Supplementary material.

### 2.10 ABC transporter analysis

The sequence of the resulting ABC transporter was analyzed using the HMMERSEARCH software of the Pfam domain database, identifying all proteins from NJ and DB that contained an ABC transporter with *P* < 0.05. The gene IDs encoding the ABC transporter were obtained, and the CDS regions of the genes responding in NJ and DB were extracted with TBtools and translated into protein sequences (Chen et al., 2020). Phylogenetic trees were constructed using MEGA 7.0 and NJ and DB’s ABC transporter sequences. The gene IDs were aligned to determine subgene families in NCBI’s Conserved Domain Database, using *Arabidopsis* and IT97K-499-35 as references. Mutated genes in the pan-genome were determined based on gene IDs.

### 2.11 Linkage map construction

The modified logarithm of odds (MLOD) scores between markers were calculated to confirm the robustness of the markers for each linkage groups (LGs). Markers with MLOD scores < 5 were filtered before ordering. To ensure efficient construction of the high-density and high-quality map, a newly developed HighMap strategy was used to order the SLAF markers and correct genotyping errors within LGs (Liu et al., 2014). Map distances were estimated using the Kosambi mapping function. References and software data are supplied as Supplementary material.

## 3 Results

### 3.1 Genome assembly

The predicted genome sizes of NJ and DB were 479.14 and 494.50 Mb, respectively (**Supplementary Figure 1**). We obtained 77 contigs with a total size of 550.31 Mb for NJ and 129 contigs with a length of 564.12 Mb for DB, and sequencing depths of 31.33 X and 32.95 X, respectively (**Table 1**). The N50 contig is 24.19 Mb for NJ and 27.56 Mb for DB (**Table 1**). When assessing the integrity of the genome assembly, 98.69% and 98.69% of the CEGMA genes and 96.28% and 96.10% of the BUSCO conserved single-copy genes were present in NJ and DB, respectively (**Supplementary Table 1**). The completeness of the assembled genome of NJ and DB is 99.16% and 98.96% when assessed by comparison with the IT97K-499-35 genome (**Table 1**, **Supplementary Table 1**). We then linked the contigs into scaffolds based on Hi-C data, NJ and DB yielded approximately 105.32 G and 212.23 G clean data, respectively (**Supplementary Table 2**). In the assembled NJ genome, 549.09 Mb sequences can be mapped on 11 chromosomes and the sequence length determining the sequence and direction is ~546.82 Mb, accounting for 99.59% of the total mapped length (**Table 1**). In DB, ~558.07 Mb of sequences can be mapped to 11 chromosomes. The sequence length that determines the sequence and direction is ~545.77 Mb, accounting for 97.80% of the total mapped length (**Table 1**). Finally, we obtained 46 scaffolds in NJ, with a scaffold N50 size of 49.11 Mb and GC content of 33.41%. In DB, 105 scaffolds with a scaffold N50 size of 48.66 Mb and GC content of 33.86% were obtained (**Table 1**). The heatmaps of the Hi-C assembled chromosomes for NJ and DB showed that the interaction strength between adjacent sequences (diagonal positions) was high. The interaction signal strength between the non-adjacent sequences (off-diagonal positions) was weak (**Supplementary Figure 2**). From the circle, we observed that the GC content on each chromosome was inversely proportional to transposable elements (TEs) and gene density, and TE density was proportional to gene density (**Supplementary Figure 3**). Collectively, the results support a high-quality assembly for the two asparagus bean genomes.

**Table 1:** Assembly features of the NJ and DB genomes.

Genome feature	NJ	DB
Total Assembly length (Mb)	550.31	564.12
Total Contig number (> 1 Kb)	77	129
Contig N50 size (bp)	24,188,537	27,857,029
Total Scaffold number (> 1 Kb)	46	105
Scaffold N50 size (bp)	49,109,670	48,657,559
GC content (%)	33.41	33.86
Gap length (Gap number)	3,100	2,400
Percent assembly anchored to 11	99.78%	98.93%
Pseudogene	71	77
Repeat sequence (%)	41.19	41.41
Number of protein-coding genes	28,370	28,425
miRNA	129	131
tRNA	1,657	1730
rRNA	6,756	9,165

### 3.2 Genome annotation

Approximately 226.70 Mb of TEs were found in the NJ genome, accounting for 41.20% of the genome (**Supplementary Table 3**), and ~233.57 Mb in DB, accounting for 41.41% of the genome (**Supplementary Table 4**). Additionally, the LTR retrotransposons of NJ and DB contained ~2.5-fold more elements in the Gypsy superfamily than Copia elements and about 1.5-fold more in IT97K-499-35. In the IT97K-499-35, CATA (5.7%) and hAT (2.4%) are the major groups of classical “cut-and-paste” transcripts in the DNA transposon (Lonardi et al., 2019). These two elements had the highest proportion in the NJ and DB genomes but a lower proportion than that in IT97K-499-35 (**Supplementary Table 3** and **Supplementary Table 4**). Approximately 60.52 and 73.59 Mb of tandem duplications were identified in the NJ and DB genomes, accounting for 11.00% and 13.50% of the genomes, respectively (**Supplementary Table 5**). Differences in motifs between NJ (1,279 motifs and 30,228 domains) and DB (1,283 motifs and 30,281 domains) suggest that the two genomes differ in molecular function, structural properties, and gene families (**Supplementary Table 6**).

We predicted 28,370 and 28,425 protein-coding genes in NJ and DB, respectively, and many were derived from homogeneous transcriptome and homology predictions (**Table 1**, **Supplementary Figure 4**, and **Supplementary Table 7**). We compared the gene number, exon number, CDS number, and intron number of related species (*P. vulgaris*, *V. radiata*, *V. unguiculata* (IT97K-499-35), and *G. max*) and found that NJ and DB are more closely related to *V. radiata* (**Supplementary Figure 5**). Approximately 98.27% and 97.52% of the complete BUSCO conserved genes are present in NJ and DB genomes, respectively (**Supplementary Table 8**). Approximately 92.57% and 91.77% of the transcriptome data were mapped to exons of NJ and DB, respectively, demonstrating high accuracy for our prediction model (**Supplementary Figure 6**). For the NJ and DB genomes, 96.9% of the 28,370 genes and 96.88% of the 28,425 genes could be annotated, respectively, using various databases (**Supplementary Table 9**). GO analysis revealed that the genes were annotated with functions related to cellular anatomical entity, catalytic activity, metabolic process, and cellular process (**Supplementary Figure 7**). Additionally, 1,657 tRNA, 6,756 rRNA, 129 miRNA, and 71 pseudogenes were annotated for NJ, and 1,730 tRNA, 9,165 rRNA, 131 miRNA, and 77 pseudogenes for DB (**Table 1**).

### 3.3 Evolutionary history of asparagus bean and syntenic comparison with IT97K-499-35

**Figure 1:**
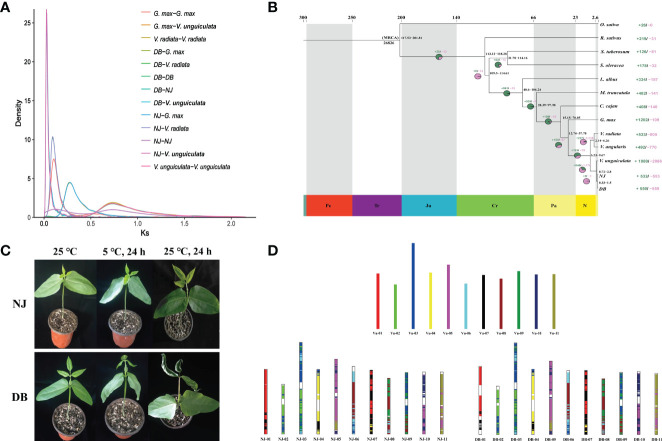
Phylogenetic relationships of asparagus bean and its genomic comparison with IT97K-499-35. **(A)**. Frequency distribution of Ks values between syntenic genes of the compared genomes. **(B)** Estimated differentiation time between asparagus bean and other plants, with O. sativa as the outgroup. Expanding and contracting gene families were identified using CAFE, and 26,826 families were deduced to exist in the most recent common ancestor (MCRA). Gene families that expand (green in the pie chart) or shrink (red in the pie chart) are plotted in the pie chart. **(C)**. Comparison of NJ and DB phenotypes under low-temperature stress (5 °C). **(D)**. Collinearity of chromosomes of NJ and DB with IT97K-499-35.

Based on 469 conserved single-copy genes, we estimated the evolutionary relationships among NJ, DB, and 11 other plants, with *O. sativa* as an outgroup (**Figure 1B**). The differentiation age between *G. max* and *V. unguiculata* was ~23.82 Mya. From the mean Ks value of the corresponding peaks (0.27), we infer a silent mutation rate (r) of 5.67 × 10^-9^ substitutions/sites/year (**Figure 1A**). Based on similar comparisons, the separation of NJ and DB from *V. unguiculata*, *V. radiata*, and *G. max* was estimated to have occurred ~4, ~16, and ~48 Mya, respectively (**Figures 1A, B**). We also estimated the divergent ages of the cowpea phylogeny, calibrating the divergent ages of the 13 species using 6 fossils with the synaptic morphology of their respective clades as crown groups. The divergence between soybean and *Vigna* occurred in the Paleogene (65−23.3 Mya) (**Figure 1B**). The whole-genome duplication (WGD) events experienced by *G. max*, *V. unguiculata*, and *V. radiata* during genome evolution were 18.25, 64.81, and 65.25 Mya, respectively (**Figure 1A**). The WGD events of DB and NJ occurred 64.55 Mya (Ks = 0.732) and 64.81 Mya (Ks = 0.735), respectively (**Figure 1A**).

**Figure 2:**
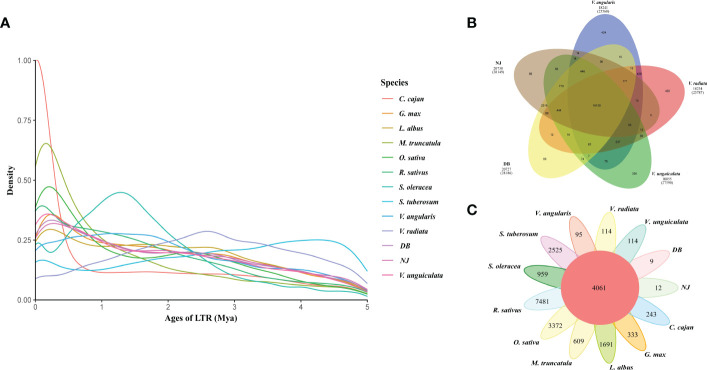
Number of orthogroups containing species and the LTR insertion time. **(A)**. LTR insertion time. The formula for calculating time is T = K/(2 × r), where the molecular clock r is chosen as 7×10^-9^. **(B)**. Venn diagram represents shared and unique gene families of five legumes (NJ, DB, *V. unguiculata*, *V. angularis*, and *V. radiata*). **(C)**. Petal map of shared and unique gene families across 13 species.

We analyzed synteny between NJ and DB and IT97K-499-35 (**Supplementary Figure 8**). There were 43,036 collinear genes in IT97K-499-35 and NJ, with collinearity of 75.92%; 43,073 genes were collinear between DB and IT97K-499-35, reaching 75.91%. From the 56,795 genes analyzed, 86.07% were collinear for the NJ and DB genomes (**Supplementary Figure 8**). The collinearity plot shows that NJ and DB have many chromosomal rearrangements compared with IT97K-499-35, and there are obvious breaks on chromosomes 01, 02, 03, 04, 05, 09, and 10 (**Figure 1D**). It was found that there was a 90.43 Mb inversion in chromosome 01 in NJ, but not in DB (**Supplementary Figure 9**). Most LTRs in the 13 species were inserted into the genome in the last 5 million years (**Figure 2A**). The pigeon bean had the earliest LTR insertion event, about 27,000 years ago, and the density of the LTR was the highest. The time of the LTR insertion event was 227,700 years ago in cowpea, 322,100 years ago in NJ, and 278,800 years ago in DB. The insertion times of the LTRs into the potato, spinach, and radish genomes were 4.25, 1.28, and 0.13 Mya, respectively (**Figure 2A**).

### 3.4 Gene family analyses

NJ and DB share many gene families with IT97K-499-35 (**Figures 2B, C**). There were 532 expansions and 553 contractions for NJ, and 550 expansions and 559 contractions for DB (**Figure 2B**). According to the GO enrichment, expanding gene families in NJ and DB may contribute to translation, metabolism and cellular process, response to stimulus, and reproduction (**Supplementary Figure 10**). Shared metabolic pathways from the KEGG analysis include photosynthesis, oxidative phosphorylation, and flavonoid biosynthesis (**Supplementary Figure 11**). In the 13 plant genomes, 44,394 orthologous gene clusters were identified, out of which 4,061 orthogroups were shared by the species. Gene family copy number differences were identified between NJ and DB (**Supplementary Figure 12**), confirming that the DB genome was larger than that of NJ. Compared with the other 13 species, NJ (12) and DB (9) had fewer species-specific orthologous gene clusters (**Figure 2C**). The Venn diagram of the gene families of NJ, DB, *V. unguiculata*, *V. angularis*, and *V. radiata* shows that the legumes share 16,130 gene families. In comparison, 69 and 66 gene families are specific to NJ and DB, respectively (**Figure 2B**). NJ species-specific gene family GO annotated terms were in response to stimulus, biological regulation, cellular component organization, or biogenesis, with only oxidative phosphorylation enriched in the KEGG database (**Supplementary Figure 13**). GO terms in DB species-specific gene families were mainly involved in transporter activity, membrane part and binding, whereas KEGG enrichment was mainly reflected in photosynthesis (**Supplementary Figure 14**).

A total of 142 genes showed positive selection in NJ. The genes belonged to several GO categories (*P* < 0.05) and KEGG pathways (*P* < 0.05), with a higher representation of RNA-related genes, such as the mRNA cleavage factor complex, RNA methyltransferase activity, and mRNA surveillance pathway (**Supplementary Table 10** and **Supplementary Table 11**). A total of 156 genes showed positive selection in DB. They were mainly enriched in DNA-dependent DNA replication, tRNA processing, and DNA repair in the biological process terms of the GO database (**Supplementary Table 10**), and in mismatch repair and DNA replication pathways in the KEGG metabolic pathway (**Supplementary Table 11**).

### 3.5 Construction of cowpea pan-genome

**Figure 3:**
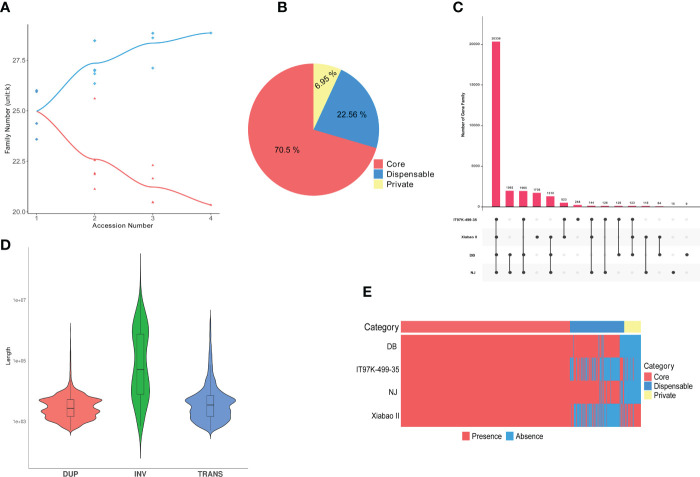
Cowpea pan-genome. **(A)**. Gene family accumulation curves for the pan-genome (blue) and core genome (orange). **(B)**. Pie charts show the proportion of each core, variable, and unique gene family. **(C)**. Quantitative dendrograms and dumbbell diagrams of core, variable, and unique gene families in the pan-genome. **(D)**. Violin plot of length statistics for each type of SV (TRANS: chromosomal translocation; INV: inversion; DUP: duplication). **(E)**. Presence/absence information of pan gene families in the NJ, DB, =IT97K-499-35 and Xiabao II.

The pan-genome was constructed using the whole-genome alignments of the four varieties (NJ, DB, IT97K-499-35 and Xiabao II). The reference genome is 550.31 Mb, the final pan genome is 616.35 Mb, and the core genome is 399.78 Mb. Statistical analysis of core and pan gene families of the four varieties showed that the number of core gene families decreased gradually with an increase in the number of species, and the number of gene families in the pan-genome increased with an increase of the number of varieties (**Figure 3A, B**). In the pan-genome, with NJ as the reference, core gene families accounted for 70.5%, variable gene families for 22.56%, and unique gene families for 6.95% (**Figure 3B**). Through functional analysis, they contained 20,336, 6,507, and 2,004 gene families, respectively. NJ and DB have similar numbers of core and variable gene families, and Xiabao II and IT97K-499-35 have similar results. However, there are large differences among the four varieties in the number of unique gene families, including 9,259 in Xiabao II, 1,751 in IT97K-499-35, 28 in DB and 53 in NJ (**Supplementary Table 12**). The UpSet figure is used to show the clustering results of gene families within four species, including 1,736 in Xiabao II, 244 in IT97K-499-35, 9 in DB and 12 in NJ (**Figure 3C**). The KEGG analysis was carried out for the unique gene families of four varieties. Xiabao II was mainly enriched in zeatin biosynthesis and propoate metropolis, IT97K-499-35 was mainly enriched in dieterpenoid biosynthesis and linoleic acid metropolis, DB was mainly enriched in inorganic animion exchange activity and calcium-dependent phospholipid binding, and in the unique gene family of NJ, it is mainly enriched in photogenic electron transport in photosystem and response to herbicide (**Supplementary Figure 15**).

### 3.6 Sequence variation analyses in the pan-genome

The three genomes (Xiabao II, IT97K-499-35 and DB) assemblies were blasted against the NJ genome to detect variations. Between each pair of genomes, 8.74×10^5^ (155,245 genes involved) single-nucleotide polymorphisms (SNPs) for Xiabao II, 1.62×10^6^ (432,899 genes involved) SNPs for IT97K-499-35, and 1.54×10^6^ (294,414 genes involved) SNPs for DB (**Supplementary Table 13**). The location information of the mutation sites on the Xiabao II, IT97K-499-35 and DB genome showed that the upstream 1 kb region had more SNPs than the UTR-5’ and the downstream 1 kb region (**Supplementary Table 14**). More SNPs were observed in the introns than the exons (**Supplementary Table 14**). Exons are the main carriers of gene function, but SNPs can cause various gene mutations, such as amino acid changes, loss of start sites or stop codons, or the premature generation of stop codons. The ratio of SNPs that lead to non-synonymous single nucleotide variants (SNVs) and synonymous SNV is roughly 1:1, and the effect of SNPs that lead to amino acid changes on exon function is mainly focused on the mutation generating stop codons (**Supplementary Table 15**). We observed 2−50 bp variant insertions and deletions (InDels) including 307,078 insertions and 107,962 deletions in Xiabao II, 245,319 insertions and 272,218 deletions in IT97K-499-35, and 175,558 insertions and 157,924 deletions in DB. Structural annotation of InDels followed a trend similar to that of the SNP structural annotation (**Supplementary Table 14**). The ratio of frameshift mutation and non-frameshift mutation caused by InDels in DB is close to 1:1, and the number of stop codons generated at the mutation site is greater than the sum of the promoter and terminator elimination caused by mutations (**Supplementary Table 15**). In Xiabao II and IT97K-499-35, InDels caused more frameshift mutations than non-frameshift mutations (**Supplementary Table 15**). In the circle plot, the frequencies of the SNPs and InDels on 11 chromosomes are similar, but their relationships with gene density are partially negatively correlated (**Supplementary Figure 16**).

There were 9,516 “presence” variants and 8,199 “absence” variants in the pan-genome (**Supplementary Table 16**). The numbers of presence/absence variants (PAVs) of Xiabao II and IT97K-499-35 were all higher than those of DB (**Supplementary Table 16**, **Figure 3E**). The PAV-affected genes are enriched in biological processes related to biotic and abiotic stress, such as response to stimulus, immune system process, defense response, signal transduction, and biological regulation (**Supplementary Table 17**).

Large-scale structural variations (SVs, 50 bp), including chromosomal inversions (INVs), chromosomal translocations (TRANs), and tandem duplications (DUPs, **Figure 3D**), are abundant in the genomes of Xiabao II and IT97K-499-35. A total of 209 INVs, 3,374 TRANs, and 12,209 DUPs were discovered (**Supplementary Table 16**). Genes affected by SVs are represented in the KEGG database by ribosome, translation, photosynthesis, oxidative phosphorylation, chloroplast thylakoid membrane, and NADH dehydrogenase (ubiquinone) activity terms (**Supplementary Figure 17**). CNVs variation is attributed to genome rearrangements, which generally refers to an increase or decrease in copy numbers of large genome fragments with a length > 1 kb (**Supplementary Table 16**). Such genes were enriched in response to stimulus, cellular component organization or biogenesis, detoxification, pentose and glucuronate interconversions, ABC transporters, and other terms (**Supplementary Figure 18**).

### 3.7 Variation in ABC transporters

**Figure 4:**
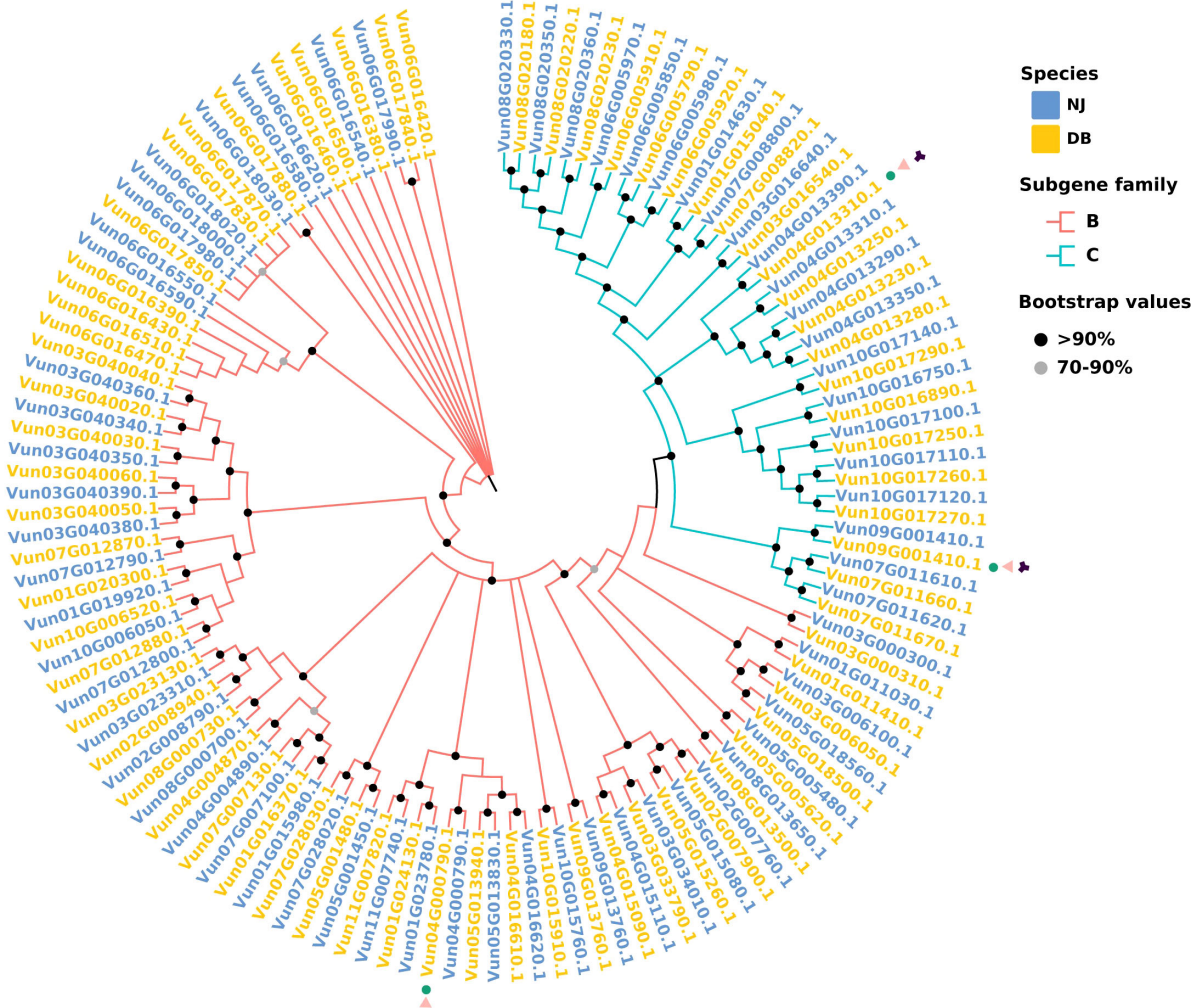
Phylogenetics of the NJ and DB ABC transporter gene families. Neighbor-joining NB-ARC tree containing gene models from both species (*P* < 0.05). The red branch represents the B subgene family of the ABC transporter, and the blue branch represents the C subgene family. The red squares represent the NJ gene model, and the blue squares represent the DB gene model. In the outer circle of the phylogenetic tree, green circles represent SNP mutations in the pan-genome, =pink triangles represent InDel mutations and black diamonds represent SV mutations.

There were 74 and 109 SNP-induced and InDel-induced ABC transporters, respectively. The number of ABC transporters affected by large fragments, such as SVs and CNVs, was relatively low, at 2 (including *ABCC* and *ABCG*) and 3 (including *ABCB* and *ABCC*), respectively. There were 7 ABC transporter subgene families (*ABC*) with “presence” variants and two (*ABCC*) with “absence” variants in the NJ *vs*. DB genomes. The genes encoding ABC transporters in the NJ and DB genomes with *P* < 0.05 were used to construct an evolutionary tree (**Figure 4**). These genes were clustered in the B and C subgene families, and SNP, InDel and SV variants in the genes were analyzed. In the DB genome, Vun04G000790.1 contains SNP and InDel mutations, Vun04G013310.1 and Vun09G001410.1 both have SNP and InDel mutations, and also have SV mutations. Phylogenetic tree shows that Vun04G000790.1 belongs to B subgene family, and Vun04G013310.1 and Vun09G001410.1 belong to C subgene family.

### 3.8 High-density genetic map construction

**Figure 5:**
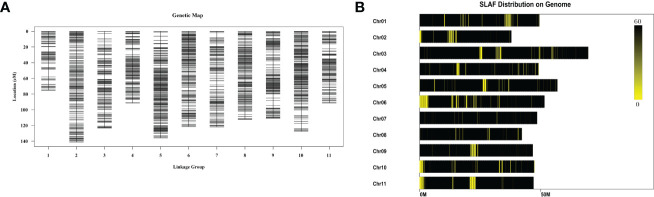
Genetic linkage map. **(A)**. Genetic map. The 9,986 SLAF tags screened out were divided into 11 linkage groups by mapping with the reference genome, and the tags whose MLOD values between the two tags were both > 3 were filtered out, and a total of 4,257 tags were markers. Each chromosome is a linkage group, and the linkage group is used as the unit to map according to the size of the linkage group map distance. **(B)**. Distribution of SLAF tags across chromosomes. The abscissa is the length of the chromosome, each yellow band represents a chromosome, and the genome is divided according to the size of 1M. The darker the area in the figure is the area where the SLAF label is concentrated.

NJ, DB, and 100 F_2_ generations, were analyzed using the locus-specific amplified fragment (SLAF) sequencing strategy. A total of 345.25 Mb of clean reads were generated, and an average of 92.58% of the reads had a Q30 quality score, indicating good sequencing quality. We obtained 284,020 SLAF tags, including 29,893 (10.52%) polymorphic SLAF tags. The average sequencing depth of the SLAF-tagged parents was 26.31 ×, and the average sequencing depth of progeny was 9.32 ×. After filtering, 9,986 SLAF tags were obtained to construct 11 linkage groups. After filtering out the MLOD values of each pair of labels below 3, the number of markers was 4,257. The result was a total map distance of 1,253.11 cM and an average genetic distance of 0.3 cM (**Figure 5A**). The number of SLAF-tags on each chromosome is shown in **Figure 5B**, **Supplementary Table 18** and **Supplementary Table 19**. We obtained 7,539 SNP markers in the graph; 2,331 SNPs were transitions, and 5,208 SNPs were transversions (**Supplementary Table 20**). The heat map shows that the linkage relationship between adjacent markers in each group is very strong, and the linkage relationship between markers weakens gradually with an increase in distance (**Supplementary Figure 19**).

## 4 Discussion

Asparagus beans are heat-, drought-, and cold tolerant. NJ has better cold resistance than DB in the seedling stage, whereas the opposite is true in the mature stage (Miao et al., 2022); therefore, NJ and DB can be used as relatively extreme materials for studying the cold resistance of asparagus beans. Through the assembly, evaluation, and analysis of the genomes of the two asparagus beans, we inferred the origin of the asparagus bean, its time of differentiation, the timing of WGD, and the rearrangement of the 11 chromosomes. The genome of DB is larger than NJ can be explained from the number and annotation of tandem repeats, this is similar to the conclusion of Dai et al. (2022). We observed that genes that improve cold tolerance were greatly expanded through gene family expansion and positive selection, improving NJ’s capacity to adapt to cold environments. Although much genetic information can be obtained from a single genome, analysis of the impact of intra-genome variation on species diversity and agronomic traits is limited (Pinosio et al., 2016; Dolatabadian et al., 2020). The construction of the pan-genome enabled us to unravel the sequence variation within the germplasm and identify genomic regions associated with temperature-adaptive traits. The results enhance our understanding of genome evolution and provide clues for genome variation and cold adaptation in asparagus beans from a pan-genome perspective.

### 4.1 Evolutionary research on asparagus beans

The assembly, annotation, and comparative genomics analyses showed that soybean and cowpea diverged ~23.82 Mya, and the fossil divergence time demarcated its origin in the Neogene period, ~23 Mya. The timings of WGD events for the NJ and DB genomes were similar to those for the *V. unguiculata* and *V. radiata* genomes (~60 Mya). A peak with a Ks value close to 0 was observed in NJ, which should be the peak generated by the increase of collinear gene pairs caused by the insertion of long fragments. Using *Arabidopsis thaliana* as an outgroup, Xu et al. (2020) found a relatively new polyploid event in legumes, a WGD event (~8.62 Mya) shared by *G. max* and its wild relative *Glycine soja*. The WGD event for *G. max* in our research occurred at ~18.25 Mya. The large gaps are probably due to the species used in the phylogenetic tree and outgroup. Using *A. thaliana* as outgroup, a phylogenetic tree showed that cowpea and soybean diverged ~21.20 Mya (Moghaddam et al., 2021); similar to our results (~23.82 Mya). Our data included spinach, radish, and potato to the evolutionary tree. However, this increased the breadth of species comparison, which may allow for relatively large time gaps in species divergence. By elucidating the insertion timing of plant-enriched LTRs, genome expansion can be timed, providing an important basis for species differentiation. It may be that the divergence time with IT97K-499-35 genome is limited, and the expansion and contraction frequency of NJ and DB gene families are also limited.

### 4.2 NJ adaptation to cold

Changes in the functions of cell membrane enzyme systems are the main manifestations of plant chilling damage (Zhu, 2016). Cold stress reduces the photosynthesis capacity of plants, reduces aerobic respiration, increases anaerobic respiration, accelerates the decomposition of proteins, causes protein deficiency and the accumulation of toxic hydrolysates, and ultimately seriously affects plant growth and development (Guo et al., 2018). In our previous study, NJ outperformed DB at 5°C, and NJ showed less low-temperature damage (**Figure 1C**). By functionally enriching the unique NJ and DB gene families that underwent expansion and positive selection, we found more gene families related to environmental fitness clustered in NJ. They included oxidative phosphorylation, flavonoid synthesis, and photosynthesis genes in the KEGG database. GO terms included responses to hydrogen peroxide, reactive oxygen species, accumulate of toxic substances, defensive responses, and ATP synthesis. Such species-specific gene functions enhance NJ’s adaptation to low-temperature environments.

### 4.3 Pan-genome construction

The asparagus bean genome and comparative genomics with other species have shown that the genetic variation information for the species has not been captured comprehensively, suggesting the need to construct a pan-genome. In addition to the conservation of the core genes (70.50%), there was diversity in the dispensable genes (22.56%) and species-specific genes (6.95%) across the pan-genome, which can facilitate the discovery of new genes to significantly improve the reference genome. Compared with, for example, white lupin (Hufnagel et al., 2021), pigeon pea (Zhao et al., 2020), soybean (Torkamaneh et al., 2021), eggplant (Barchi et al., 2021), cotton (Li et al., 2021), and rice (Qin et al., 2021), the pan-genome of cowpea has a high content of core genes, which may reflect its short domestication history. Functional analysis of the dispensable genes showed their association with enhanced stress adaptation, signal transduction, and seed storage. *Vigna* is an adaptable and resilient crop variety, and current breeding programs aim to enhance food security in the wake of climate change risks. The functional enrichment of such variable genes could facilitate the achievement of this aim (Vincent et al., 2013; Zuluaga et al., 2021).

### 4.4 Functional analysis of variant genes in the pan-genome

Small variants (SNPs and InDels) and large structural variants (SVs, PAVs, and CNVs) are central to the pan-genome. Among the variants caused by SNPs and InDels, many genes have similar functions. The *ICE1-CBF-COR* is a classic cold response pathway. *ICE1* (SNP/InDel-affected) is an MYC-type bHLH transcription factor that can activate *DREB/CBFs* (SNP/InDel-affected). This reaction up-regulates the downstream cold stress-related *COR* (InDel-affected) genes and ultimately improves the tolerance response of asparagus bean to cold signals and stress (**Supplementary Table 21**). Small variations have also been found in genes associated with flowering, such as the RNA-binding protein FCA, encoding a strong promoter for the transition to flowering in Arabidopsis (Wang et al., 2020), that exhibits SNP variation and is associated with early flowering-related gene protein ELF4-Like, which exhibits InDel variations (Lin et al., 2019) (**Supplementary Table 16**). The central roles of heat stress transcription factor and drought-inducible protein 19 (Liu et al., 2013) under various abiotic stresses are influenced by SNPs and InDels, respectively, influencing the drought tolerance of asparagus bean. Numerous helix-loop-helix DNA-binding domain genes were found in the SNP and InDel variants, many encoding phytochrome-interacting factors (*PIFs*). Studies in *A. thaliana* found that *PIFs* play a central role in plant photomorphogenesis, inhibiting seed germination and regulating photogenesis in seedlings. It was also shown that abiotic stress and hormonal signal pathways converge to regulate *PIF* activity (Wang et al., 2022). The researchers also pointed out that the role of the light-induced signal network and the related molecular mechanisms will promote future research on light signal transcription factors and stress tolerance in other crops (Wang et al., 2020). This information should enable breeders to increase asparagus bean yields and develop climate-tolerant asparagus beans that can adapt to changing environmental conditions.

Seed color diversity is one of the main characteristics of *Vigna*, and several gene models identified proteins involved in the late regulation of the flavonoid biosynthesis pathway as candidate genes for seed color by QTL mapping, including the basic helix-loop-helix gene at the C locus, the WD-repeat gene, and the E3 ubiquitin ligase gene (Herniter et al., 2019). When comparing NJ as a reference, we did not find any genes related to flavonoid synthesis in the PAVs. These genes may be highly conserved in *Vigna*, but genes encoding the E3 ubiquitin-protein ligases that may indirectly affect the accumulation of seed coat color were found in the PAVs. Some PAV-affected genes have key roles in cold and heat stress and important agronomical traits. These include *CRPK1* and *14-3-3*, two modules that transmit cold signals from the plasma membrane to the nucleus to regulate CBF stability and, in turn, asparagus bean response to cold stress (Liu et al., 2017). Genes affected by PAV are included in the protease inhibitor/seed storage/LTP family (Meitei et al., 2018), which improves seed utilization, resulting in cowpea asparagus beans high in calories and protein.

### 4.5 Variations of the ABC transporters

ABC transporters or ATP-binding cassette proteins are an ancient and large family of ATP-driven pumps that play important roles in resistance to biotic pathogens, abiotic stress, and growth and development (Do et al., 2018). The ABC transporters are divided into eight subfamilies in plants: *ABCA-ABCG* and *ABCI*. The members of this family are involved in plant hormone transport, heavy metal ion efflux, plant response to environmental stress, and transportation of secondary metabolites (Dahuja et al., 2021). Due to the broad roles of ABC transporters, we searched on a pan-genome basis. There were *ABCA* and seed dormancy genes in DB among the “presence” variants. *ABCA* induces the accumulation of triglycerides in seeds, thereby prolonging the seed dormancy period. This partly explains that DB appeared three days later than NJ. Additionally, there were “absence” variants in the DB genome within the *ABCC* subgene family. Whose primary function is multidrug resistance-associated transporters, and which are localized in the tonoplast in pathogenic microbial responses and play important roles in secondary metabolism and can improve NJ stress resistance. These results partly explain the better stress resistance of NJ.

## 5 Conclusions

The dissection of the asparagus bean genome sequence provides insights into its evolution and cold tolerance and is an important resource for genomic sequence variation analysis. Comparative evolutionary studies suggest that genes involved in the expansion and active selection of cold stress responses and plant-pathogen interactions may facilitate the production of plants with improved environmental adaptation and enhanced agricultural traits. The analysis of the asparagus bean pan-genome provides new insights into the size of the core and dispensable genes and differences between the NJ and DB genomes. These results provide a more comprehensive and important resource for future functional studies of asparagus beans and the development of cold-tolerant legumes.

## Data availability statement

The data presented in the study are deposited in the National Center for Biotechnology Information (NCBI) BioProject database, accession number PRJNA869326.

## Author contributions

LeL made significant contributions to the conception, data analysis and interpretation. JZ, JX, XL, HT, XS, LZ, XX, LX, PZ and JR participated in critical revisions of important intellectual content and contributed to the previous material; BS, ZH, YT, LiL, GS and YL reviewed and edited the manuscript. HL provided supervision and project management. All authors contributed to the article and approved the submitted version.
