# Supplementary material for: Genome and pan-genome assembly of asparagus bean (Vigna unguiculata ssp. sesquipedialis) reveal the genetic basis of cold adaptation
Source: Front Plant Sci. 2022 Dec 16;13:1059804. doi: 10.3389/fpls.2022.1059804 (PMC9802904; doi:10.3389/fpls.2022.1059804)
Supplement: Supplementary file 2 [file DataSheet_1.pdf]

Supplementary Material

1 Supplementary Materials and methods

Genome sequencing, assembly and evaluation

We constructed Hi-C fragment libraries from ~300 bp insert size and sequenced through Illumina platform. Briefly, adapter sequences of raw reads were trimmed and low quality PE reads were removed for clean data. The clean Hi-C reads were first truncated at the putative Hi-C junctions and then the resulting trimmed reads were aligned to the assembly results with BWA aligner. Only uniquely alignable pairs reads whose mapping quality more than 20 were remained for further analysis. Invalid read pairs, including Dangling-End and Self-cycle, Re-ligation and Dumped products, were filtered by HiC-Prov2.8.1.

The short reads from the Illumina platform were quality filtered by HTQC v1.92.310 1. The quality-filtered reads were used for genome size estimation. We generated the 21-mer occurrence distribution of sequencing reads from short libraries using the k-mer method 2. The assembled genome was also subjected to BUSCO v53, and Cegma (v2.5) 4 was used to evaluate the integrity of the final genome assembly, aligning short reads from the Illumina platform to the reference genome 5. Thus, the high alignment ratio demonstrated the high quality of the contig assembly. The unique mapped read pairs were valid interaction pairs and were used for correction of scaffolds and clustered, ordered and orientated scaffolds onto chromosomes by LACHESIS. Before chromosomes assembly, we first performed a preassembly for error correction of scaffolds which required the splitting of scaffolds into segments of 50 kb on average. The Hi-C data were mapped to these segments using BWA (version 0.7.10-r789) software. The uniquely mapped data were retained to perform assembly by using LACHESIS software. Any two segments which showed inconsistent connection with information from the raw scaffold were checked manually. These corrected scaffolds were then assembled with LACHESIS. Parameters for running LACHESIS included: CLUSTER_MIN_RE_SITES = 30; CLUSTER_MAX_LINK_DENSITY = 2; ORDER_MIN_N_RES_IN_TRUNK = 71; ORDER_MIN_N_RES_IN_SHREDS = 72. After this step, placement and orientation errors exhibiting obvious discrete chromatin interaction patterns were manually adjusted. Finally, scaffolds were anchored to chromosomes.

TE annotation

We first customized a denovo repeat library of the genome using RepeatModeler, which can automatically execute two de novo repeat finding programs, including RECON (v1.08) and RepeatScout. Then full-length long terminal repeat retrotransposons (fl-LTR-RTs) were identified using both LTRharvest (-minlenltr 100

-maxlenltr 40000 -mintsd 4 -maxtsd 6 -motif TGCA -motifmis 1 -similar 85 -vic 10 -seed 20 -seqids yes) and LTR_finder (-D 40000 -d 100 -L 9000 -l 50 -p 20 -C -M 0.9). The high-quality intact fl-LTR-RTs and non-redundant LTR library were then produced by LTR_retriever. Non-redundant species-specific TE library was constructed by combining the denovo TE sequences library above with the known Repbase (v19.06), REXdb (V3.0) and Dfam (v3.2) database. Final TE sequences in the xx genome were identified and classified by homology search against the library using RepeatMasker v4.10. Tandem repeats were annotated by Tandem Repeats Finder and MIcroSAtellite identification tool (MISA v2.1).

Gene prediction and annotation

We integrated three approaches, namely, de novo prediction 6: Augustus (version 2.4) and SNAP(2006-07-28), homology search 7: GeMoMa (v1.7) software, and transcript-based assembly 8: RNA-sequencing data were mapped to the reference genome using Hisat (v2.0.4) and assembled by Stringtie (v1.2.3), to annotate protein-coding genes in the genome. GeneMarkS-T (v5.1) was used to predict genes based on the assembled transcripts. The PASA (v2.0.2) software was used to predict genes based on the unigenes (and full-length transcripts from the PacBio (ONT) sequencing) assembled by Trinity (v2.11). Gene models from these different approaches were combined using the EVM software (v1.1.1) and updated by PASA. The final gene models were annotated by searching the GenBank Non-Redundant (NR, 20200921), TrEMBL (202005), Pfam (33.1), SwissProt (202005), gene ontology (GO, 20200615) and Kyoto Encyclopedia of Genes and Genomes (KEGG, 20191220) databases.

Annotation of non-coding RNA and Pseudogenes

We used tRNAscan-SE (v2.0.9) 9 algorithms with default parameters to identify the genes associated with tRNA and used Barrnap (v0.9) with default parameters to identify the genes related to rRNA. MiRNAs and snRNAs were identified by Infernal (v1.1.1) 10software against the Rfam (v14.5) database with default parameters. The GenBlastA (v1.0.4) 11 program was used to scan the whole genomes after masking predicted functional genes. Putative candidates were then analyzed by searching for non-mature and frame-shift mutations using GeneWise (v2.4.1) 12.

Gene family identification

The protein-coding sequences in the NJ and DB genome and genomes from 11 other species were compared using OrthoFinder (v2.4.0) 13 software. The PANTHER V15 14 database was used to annotate the gene families and perform GO and KEGG enrichment analysis for this species-specific gene family.

Phylogenetic analysis

The protein sequences of the 469 single-copy orthologous genes were aligned with the MAFFT (v7.205) program 15, and the corresponding CDS alignments were generated and concatenated with the guidance of protein alignment. IQ-TREE (v1.6.11) was used to construct the phylogenetic tree 16. The phylogenetic relationship of other closely related species was consistent with previous studies. We set the outgroup as O. sativa and then used the software package MCMCTREE that comes with the PAML v4.9i software to calculate the divergence time 17. The final evolutionary tree with differentiation time was obtained graphically using MCMCTreeR v1.118.

Gene family expansion and contraction analysis

Based on the identified gene families and the constructed phylogenetic tree with predicted divergence times, we used CAFÉ to analyze the expansion and contraction of gene families 19. The criteria for significant expansion or contraction of a gene family is that both family-wide P-Values and viterbi P-Values are less than 0.05. GO, and KEGG enrichment analysis was performed on the expansion and contraction gene families of NJ and DB.

Positively selected genes

We mainly use the CodeML module in PAML for positive selection analysis. Specifically, to obtain single-copy gene families between V. angularis, V. radiata, V. unguiculata, DB and NJ, and then use MAFFT (parameter: --localpair --maxiterate 1000) to compare the protein sequences of each gene family, then reversed to codon-aligned sequences using PAL2NAL, and finally used CodeML (using the F3x4 model of codon frequencies) based on the Branch-site model to pair the two models Model A (assuming the foreground branch ω) by the "chi2" program under PAML (All are in positive selection, that is, ω>1) and null Model (meaning that the ω value of any site is not allowed to be greater than 1) are subjected to likelihood ratio tests, and the results of significant differences are obtained (P <0.05), and use the Bayesian method (BEB, Bayes empirical Bayes method) to obtain the posterior probability of a site that is considered to be positively selected (usually greater than 0.95 is considered to be a site that is significantly positively selected), and finally obtain a site that is significantly positively selected. GO and KEGG enrichment analysis of positively selected genes.

Whole-genome duplication analysis

WGDs are events in which the genome is doubled. The Ks method and 4DTv methods are commonly used to identify WGDs. We used WGD (v1.1.0) 20 software and a custom script (https://github.com/JinfengChen/Scripts) to identify WGD events.

Pan-genome construction and gene family analysis

NJ was selected as the reference genome, and three accessions (NJ, DB, IT97K-499-35 and Xiabao II) were used for pan-genome construction based on de novo alignment. The genome sequences of the NJ and DB were aligned with the reference genome sequences with the software MUMmer4.0 21, and the pan-genome was constructed with the software ppsPCP 22. The protein sequences of the three species were classified by OrthoFinder v2.3.7 software 13.

Variation analysis

NJ was the reference genome, and whole-genome alignment was performed using MUMmer4.0 21, followed by SyRI (Synteny and Rearrangement Identifier) 23 to detect variants. MUMmer software package usage process: (1) Use the nucmer program to align the reference genome with the target genome, parameter --maxmatch -c 500 -b 500 -l 100 -t 6; (2) Use the delta-filter program to compare the results To filter, parameter -1 -i 90 -l 500; (3) Use the show-coords program to convert the alignment coordinates, parameter -THrd. SyRI detects variants using default parameters. SyRI identifies collinearity regions, structural rearrangements (inversions, translocations, and duplications), local variations (SNPs, InDels, SVs, PAVs, CNVs), and regions not aligned. After detecting SNPs and InDels by SyRI, they were annotated using the ANNOVAR software toolkit 24. SyRI detects SV variants (chromosomal translocation, TRANS; inversion, INV; duplication, DUP). A variant with a sequence length of more than 50 bp and not present in the reference genome is defined as a presence variant (Presence); conversely, a variant with a sequence length of more than 50 bp and only present in the reference genome is defined as a deletion variant (Absence). We performed GO and KEGG enrichment analysis for the genes where the variants are located 25.

ABC transporter analysis

The sequence of the resulting ABC transporter was analyzed using the HMMERSEARCH software of the Pfam domain database, identifying all proteins from NJ and DB that contained an ABC transporter with $P < 0.05$. The gene IDs encoding the ABC transporter were obtained, and the CDS regions of the genes responding in NJ and DB were extracted with TBtools and translated into protein sequences 26. Phylogenetic trees were constructed using MEGA 7.0 using NJ and DB’s ABC transporter sequences. The genes ID were aligned to determine subgene families in NCBI's Conserved Domain Database, with Arabidopsis and cowpea as references. Find mutated genes in the pan-genome based on genes ID.

SLAF sequencing, grouping and genotyping

The reference genome of NJ was used to design marker discovery experiments by simulating in silico the number of markers produced by different enzymes. Pair-end sequencing (each end 125 bp) was performed on an Illumina HiSeq 2500 system (Illumina, Inc; San Diego, CA). Only SLAFs with two to four alleles were identified

as polymorphic and considered potential markers. The marker coding of polymorphic SLAFs was analyzed according to the population type F2 population. The polymorphism label of the aa × bb type (homogeneity) was selected as an effective label conforming to the population characteristics, and the polymorphism was 7.17%.

Linkage map construction

Marker loci were partitioned primarily into linkage groups (LGs) based on their locations on the genome. Next, the modified logarithm of odds (MLOD) scores between markers was calculated to confirm further the robustness of the markers for each LG. Markers with MLOD scores < 5 were filtered before ordering. To ensure efficient construction of the high-density and high-quality map, a newly developed HighMap strategy was utilized to order the SLAF markers and correct genotyping errors within LGs 27. Map distances were estimated using the Kosambi mapping function.

References

1 Yang X, Liu D, Liu F et al. HTQC: A fast quality control toolkit for Illumina sequencing data. BMC Bioinformatics 2013; 14: 2–5.

2 Liu B, Shi Y, Yuan J et al. Estimation of genomic characteristics by analyzing k-mer frequency in de novo genome projects. 2013.http://arxiv.org/abs/1308.2012.

3 Simão FA, Waterhouse RM, Ioannidis P, Kriventseva E V., Zdobnov EM. BUSCO: Assessing genome assembly and annotation completeness with single-copy orthologs. Bioinformatics 2015; 31: 3210–3212.

4 Parra G, Bradnam K, Korf I. CEGMA: A pipeline to accurately annotate core genes in eukaryotic genomes. Bioinformatics 2007; 23: 1061–1067.

5 Lonardi S, Muñoz-Amatriaín M, Liang Q et al. The genome of cowpea (Vigna unguiculata [L.] Walp.). Plant J 2019; 98: 767–782.

6 Stanke M, Steinkamp R, Waack S, Morgenstern B. AUGUSTUS: A web server for gene finding in eukaryotes. Nucleic Acids Res 2004; 32: 309–312.

7 Pertea M, Pertea GM, Antonescu CM, Chang TC, Mendell JT, Salzberg SL. StringTie enables improved reconstruction of a transcriptome from RNA-seq reads. Nat Biotechnol 2015; 33: 290–295.

8 Grabherr MG, Haas BJ, Yassour M et al. Full-length transcriptome assembly from RNA-Seq data without a reference genome. Nat Biotechnol 2011; 29: 644–652.

9 Lowe TM, Eddy SR. TRNAscan-SE: A program for improved detection of transfer RNA genes in genomic sequence. Nucleic Acids Res 1996; 25: 955–964.

10 Nawrocki EP, Eddy SR. Infernal 1.1: 100-fold faster RNA homology searches. Bioinformatics 2013; 29: 2933–2935.

11 She R, Chu JSC, Wang K, Pei J, Chen N. genBlastA: Enabling BLAST to identify homologous gene sequences. Genome Res 2009; 19: 143–149.

12 Birney E, Clamp M, Durbin R. GeneWise and Genomewise. Genome Res 2004; 14: 988–995.

13 Emms DM, Kelly S. OrthoFinder: Phylogenetic orthology inference for comparative genomics. Genome Biol 2019; 20: 1–14.

14 Mi H, Muruganujan A, Ebert D, Huang X, Thomas PD. PANTHER version 14: More genomes, a new PANTHER GO-slim and improvements in enrichment analysis tools. Nucleic Acids Res 2019; 47: D419–D426.
15 Katoh K, Asimenos G, Toh H. Multiple alignment of DNA sequences with MAFFT. Methods Mol Biol 2009; 537: 39–64.
16 Nguyen LT, Schmidt HA, Von Haeseler A, Minh BQ. IQ-TREE: A fast and effective stochastic algorithm for estimating maximum-likelihood phylogenies. Mol Biol Evol 2015; 32: 268–274.
17 Yang Z. Paml: A program package for phylogenetic analysis by maximum likelihood. Bioinformatics 1997; 13: 555–556.
18 Puttick MN. MCMCtreeR: Functions to prepare MCMCtree analyses and visualize posterior ages on trees. Bioinformatics 2019; 35: 5321–5322.
19 Han M V., Thomas GWC, Lugo-Martinez J, Hahn MW. Estimating gene gain and loss rates in the presence of error in genome assembly and annotation using CAFE 3. Mol Biol Evol 2013; 30: 1987–1997.
20 Zwaenepoel A, Van De Peer Y. Wgd-simple command line tools for the analysis of ancient whole-genome duplications. Bioinformatics 2019; 35: 2153–2155.
21 Marçais G, Delcher AL, Phillippy AM, Coston R, Salzberg SL, Zimin A. MUMmer4: A fast and versatile genome alignment system. PLoS Comput Biol 2018; 14: 1–14.
22 Tahir M, Zhu X, Xing F, Chen L. Genome analysis ppsPCP : A Plant Presence / absence Variants Scanner and Pan-genome Construction Pipeline. 2019; : 2–3.
23 Goel M, Sun H, Jiao WB, Schneeberger K. SyRI: finding genomic rearrangements and local sequence differences from whole-genome assemblies. Genome Biol 2019; 20: 1–13.
24 Wang K, Li M, Hakonarson H. ANNOVAR: Functional annotation of genetic variants from high-throughput sequencing data. Nucleic Acids Res 2010; 38: 1–7.
25 Yu G, Wang LG, Han Y, He QY. ClusterProfiler: An R package for comparing biological themes among gene clusters. Omi A J Integr Biol 2012; 16: 284–287.
26 Chen C, Chen H, Zhang Y et al. TBtools: An Integrative Toolkit Developed for Interactive Analyses of Big Biological Data. Mol Plant 2020; 13: 1194–1202.
27 Liu D, Ma C, Hong W et al. Construction and analysis of high-density linkage map using high-throughput sequencing data. PLoS One 2014; 9. doi:10.1371/journal.pone.0098855.
28 Broman KW, Wu H, Sen Ś, Churchill GA. R/qtl: QTL mapping in experimental crosses. Bioinformatics 2003; 19: 889–890.

2 Supplementary Figures and Tables

2.1 Supplementary Figures

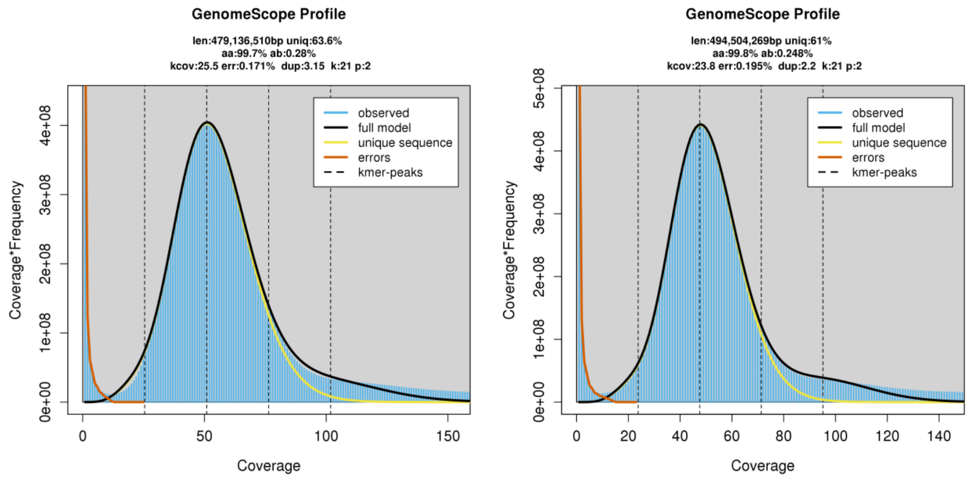

Supplementary Figure 1. Genome survey figure (NJ-left, DB-right).

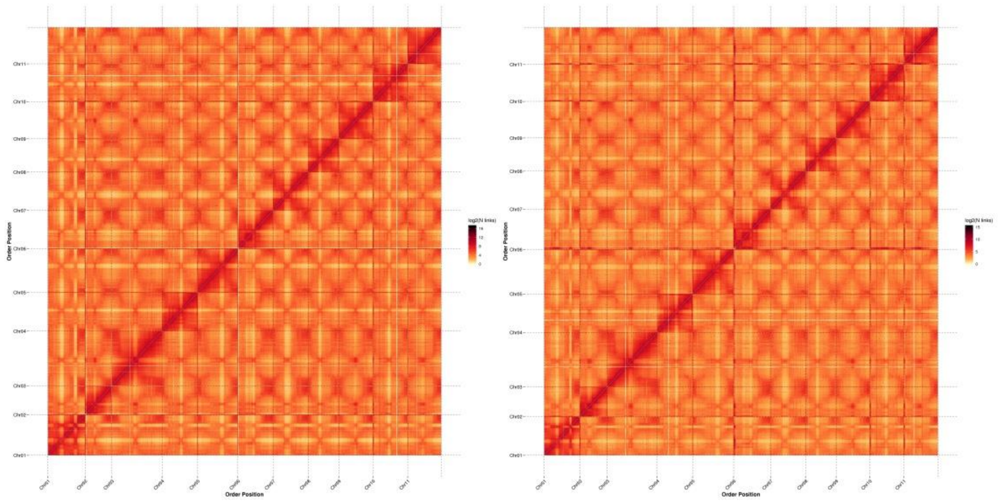

Supplementary Figure 2. Heatmap assessment of chromosomal genomes (NJ-left; DB-right).

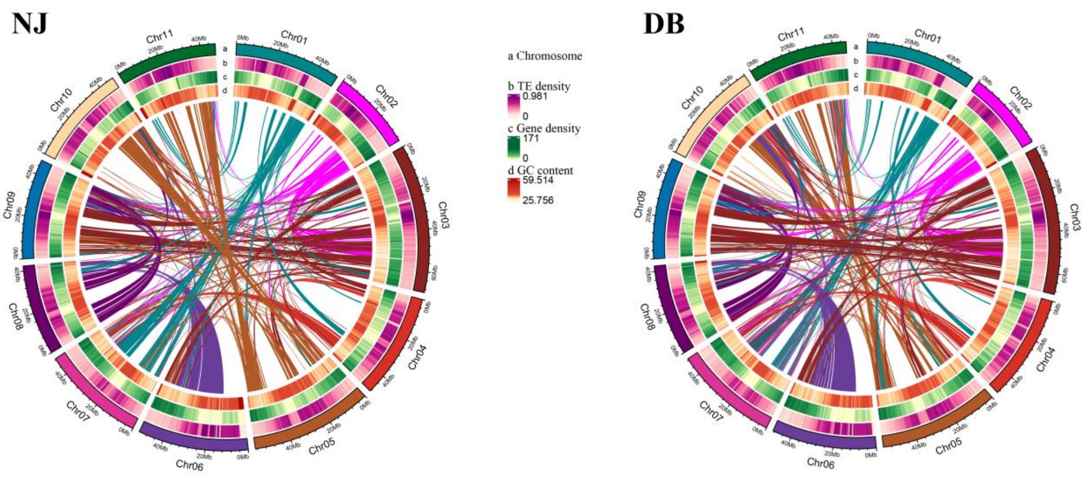

Supplementary Figure 3. Circle plots of gene density, TE density, GC content, and chromosome collinearity. The outermost circle (a) is the chromosome number; b, c, and d represent the TE density, gene density, and GC content at the corresponding chromosome position, respectively. The innermost circle is the collinearity of the 11 chromosomes in the species.

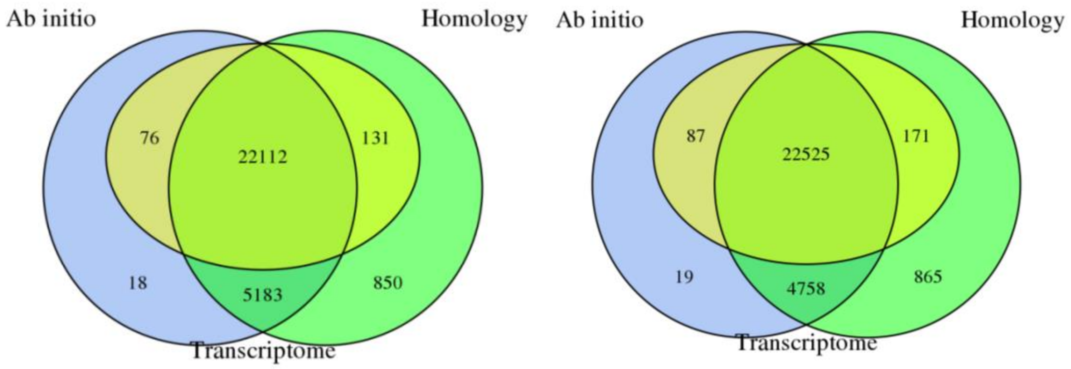

Supplementary Figure 4. Distribution map of genes derived from three prediction methods after EVM integration (NJ-Left; DB-Right).

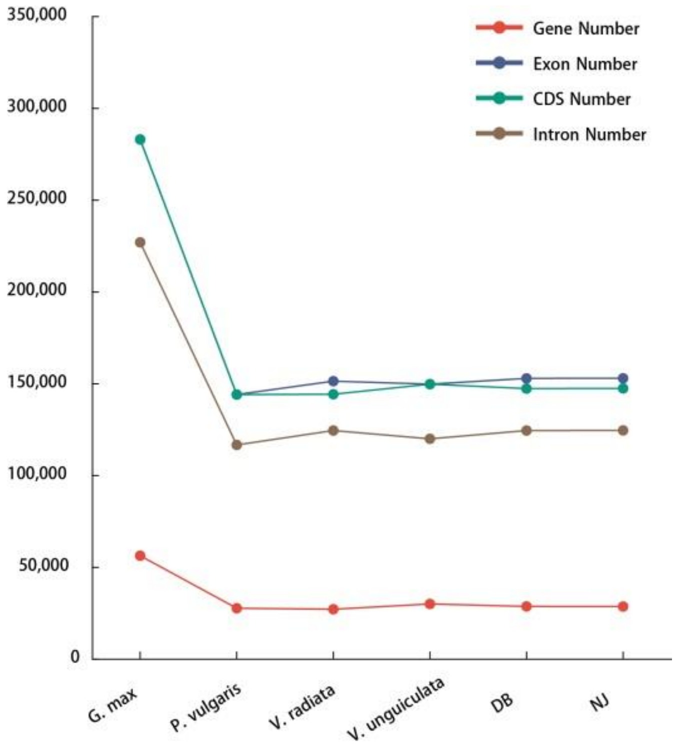

Supplementary Figure 5. Statistical information on genes encoding proteins from close species. Abscissa: Gene number, Exon Number, CDS Number, Intron Number. Different colors represent *G. max*, *P. vulgaris*, *V. radiata*, *V. unguiculata*, DB, and NJ.

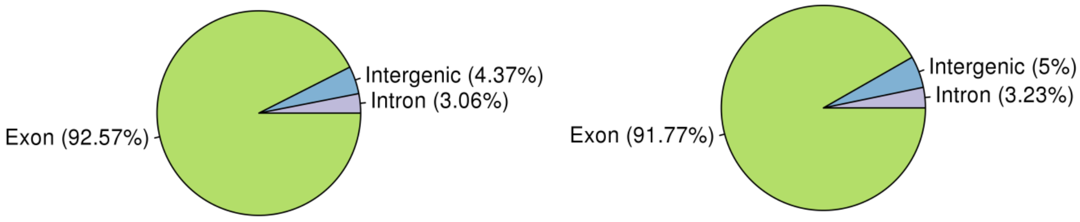

Supplementary Figure 6. Transcriptome back comparison statistics (NJ-Left; DB-Right)

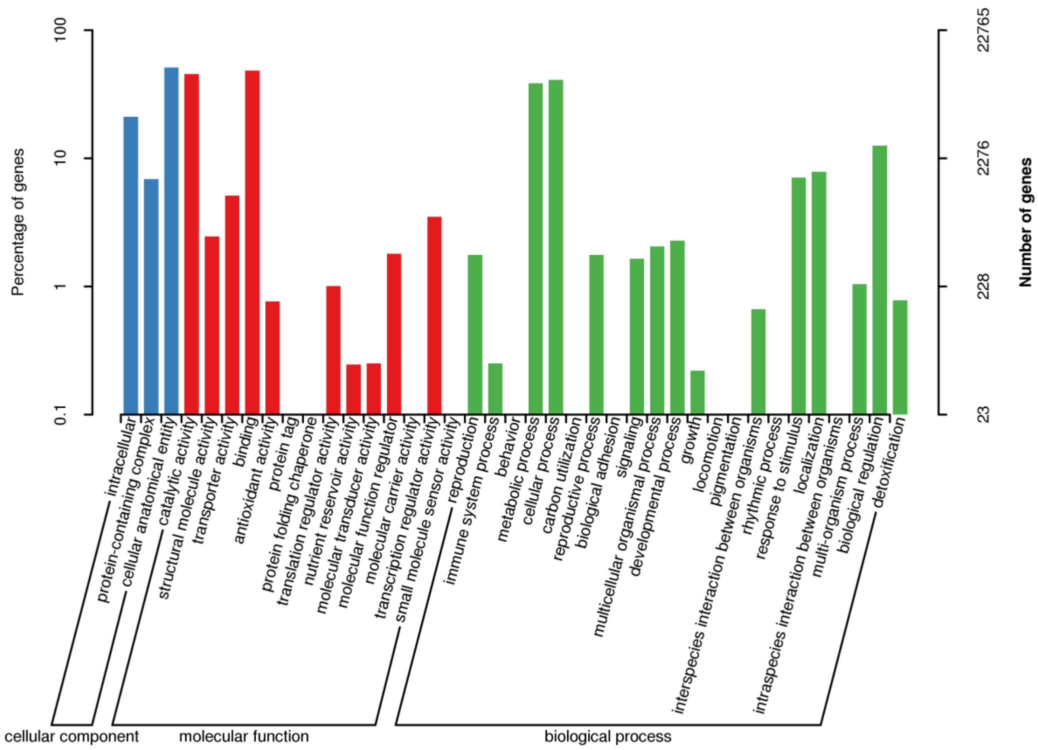

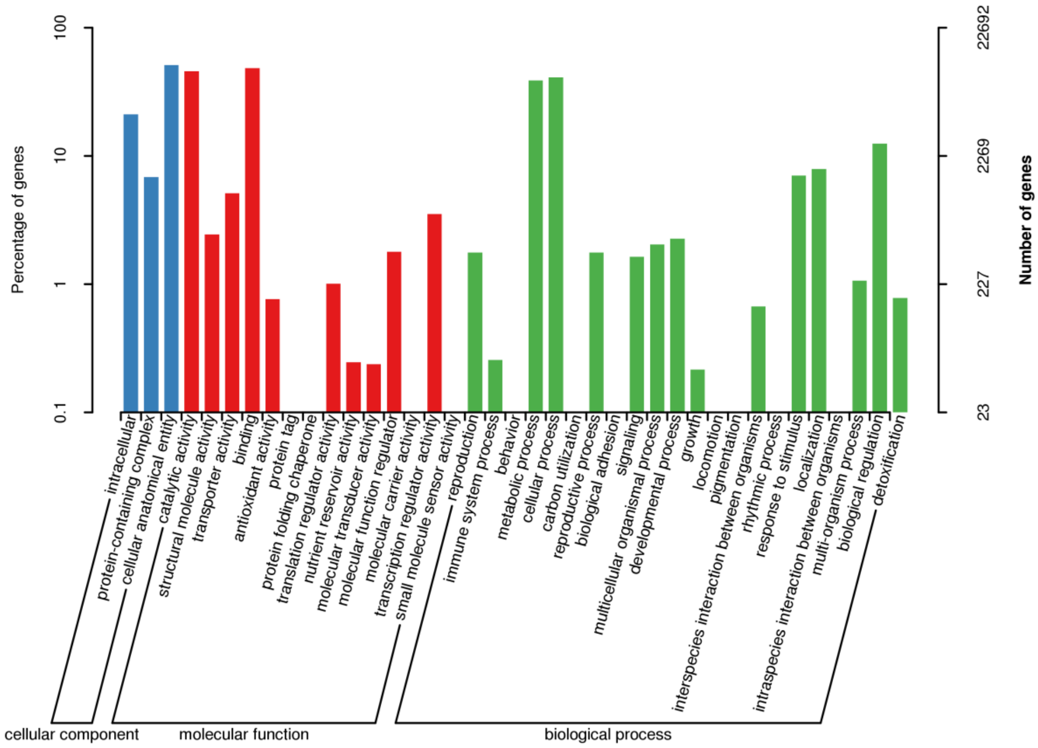

Supplementary Figure 7. GO enrichment of genes in NJ (up) and DB (down).

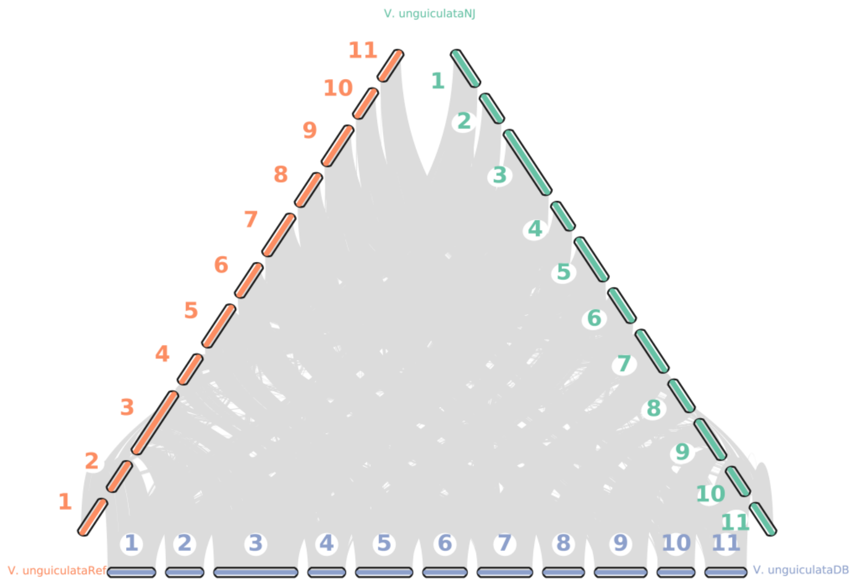

Supplementary Figure 8. Genome Collinearity for NJ_DB_ (IT97K-499-35)

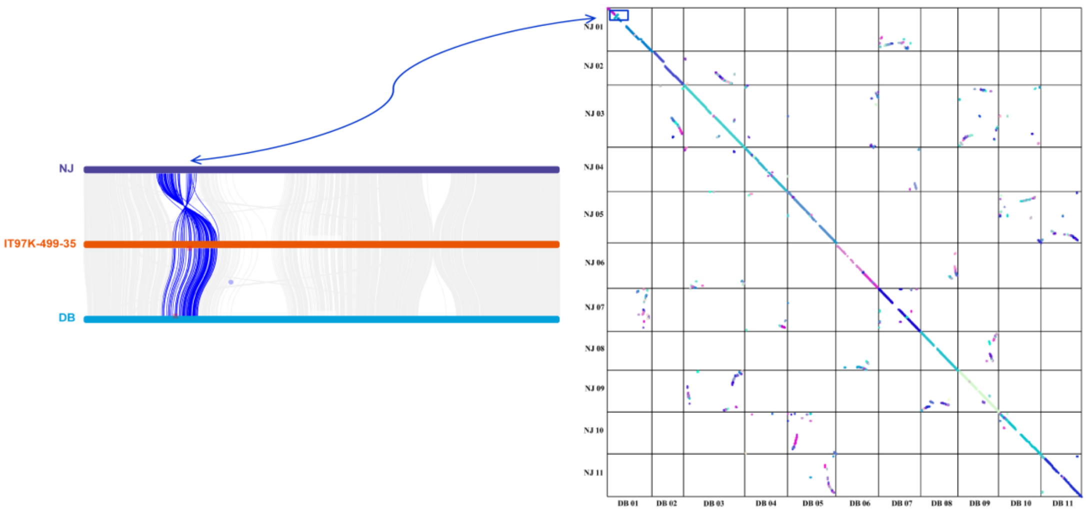

Supplementary Figure 9. Collinearity of NJ and DB chromosomes and inversion of Chr01. The collinearity of NJ and DB and a chromosomal inversion of 90.43 Mb were found on Chr01 using cowpea as the reference genome.

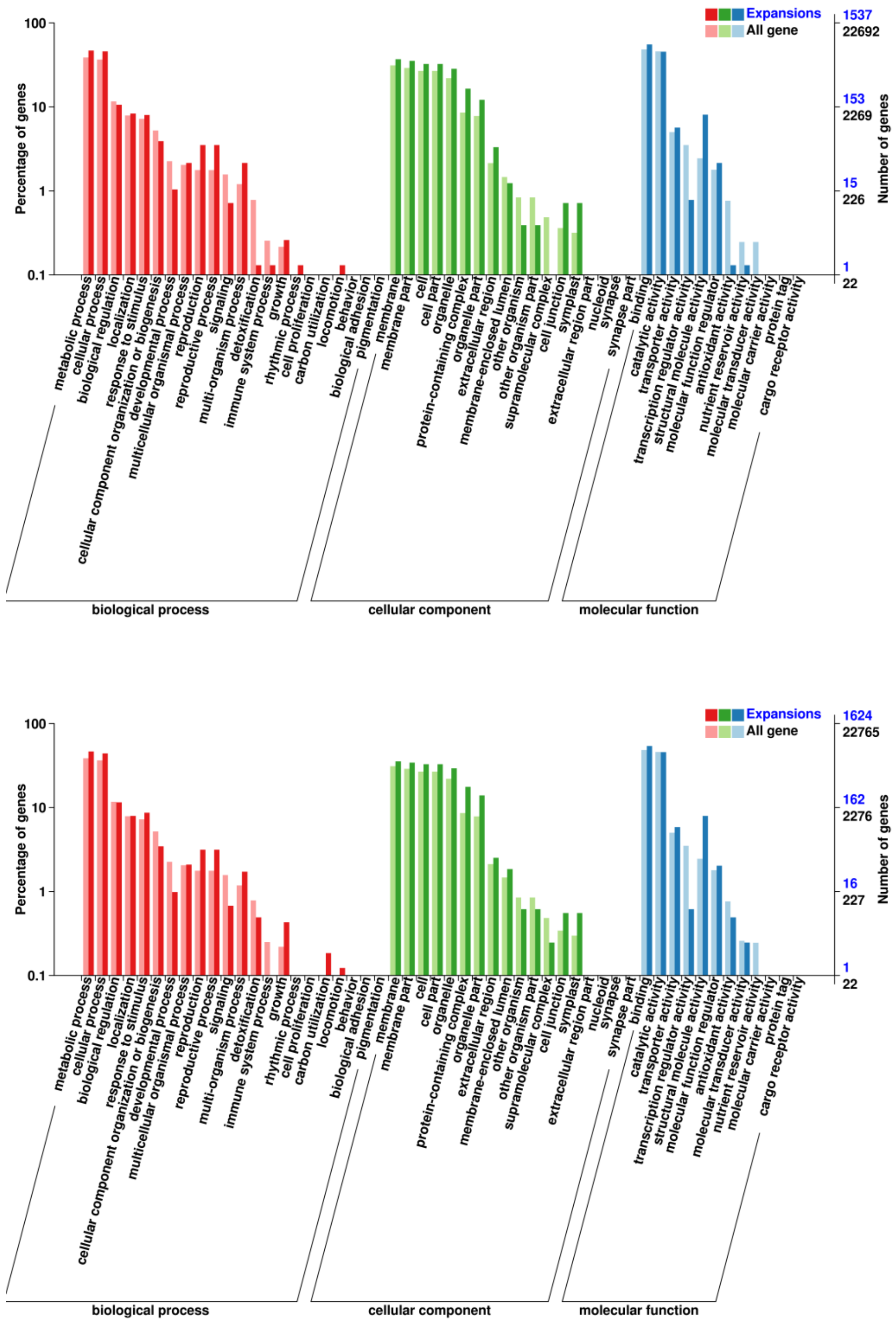

Supplementary Figure 10. GO enrichment of expanded gene families in the genome (NJ-up; DB-down)

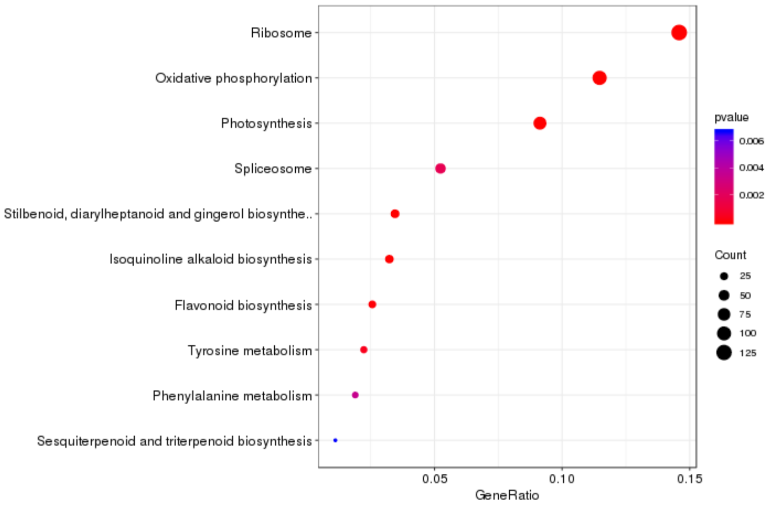

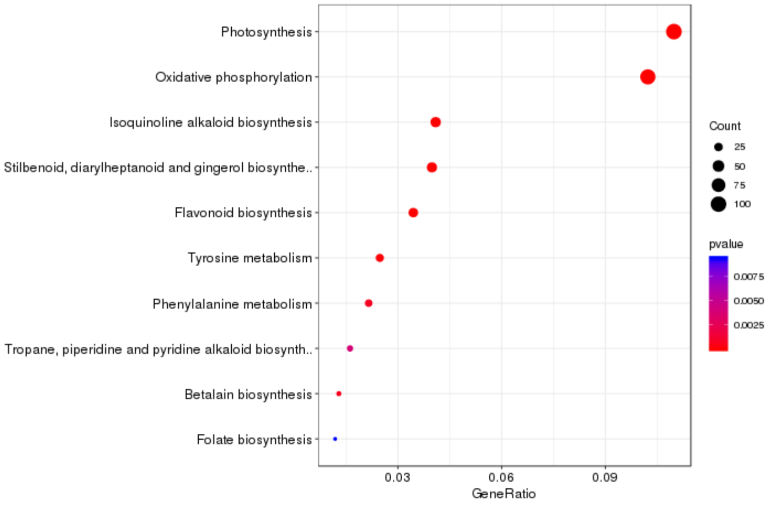

Supplementary Figure 11. KEGG enrichment of expanded gene families in the genome (NJ-up; DB-down).

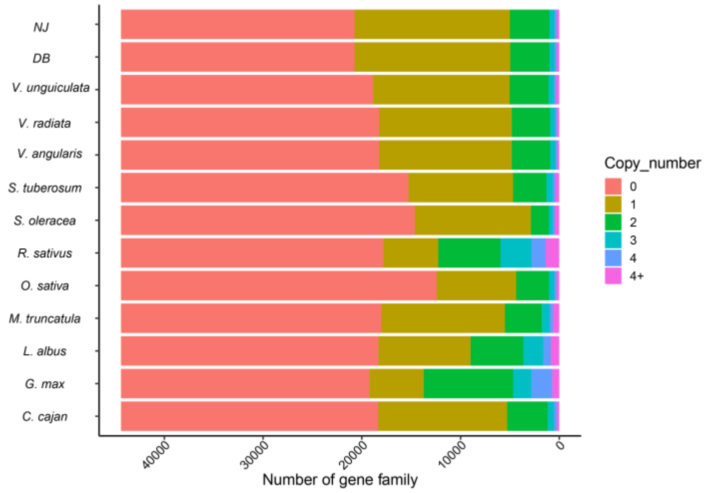

Supplementary Figure 12. Species-specific gene family copy number

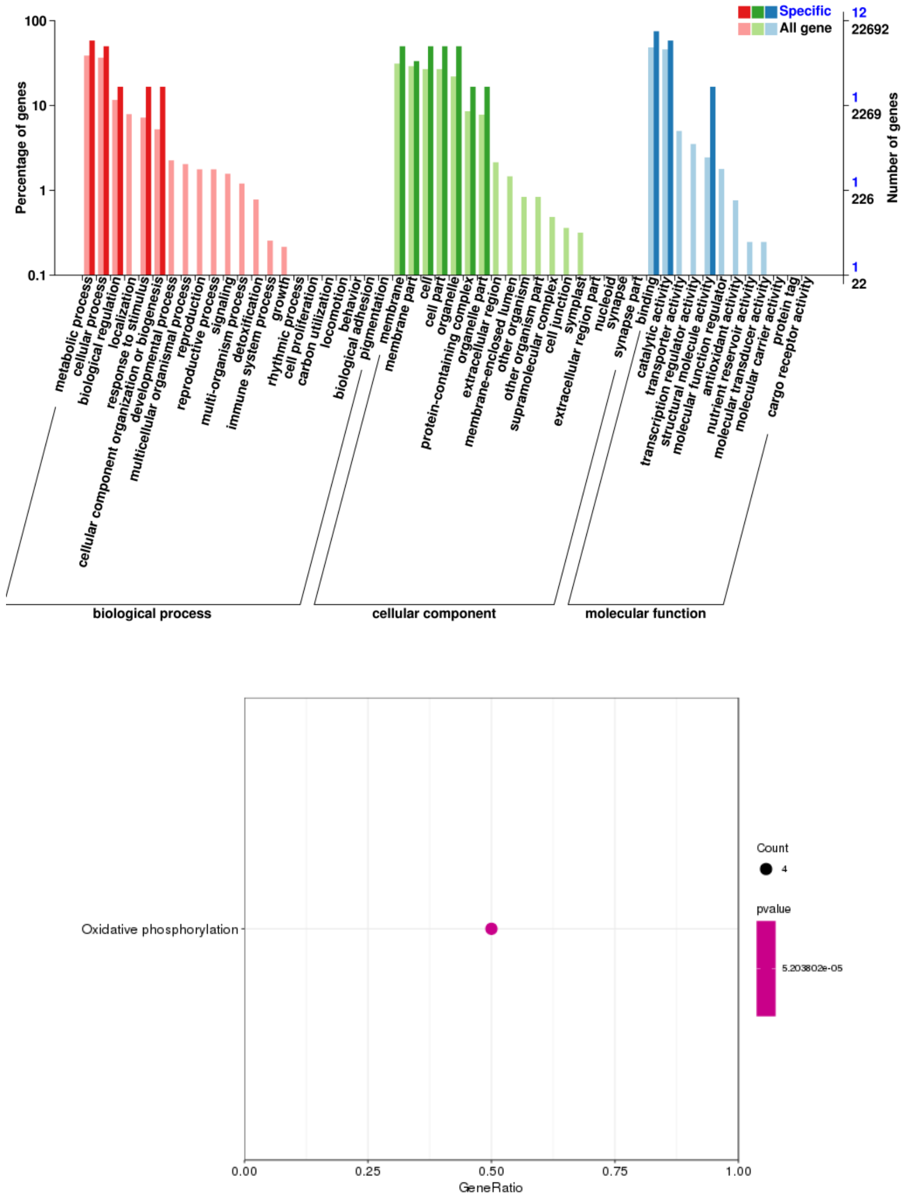

Supplementary Figure 13. Species-specific orthogroups of NJ (GO and KEGG annotation)

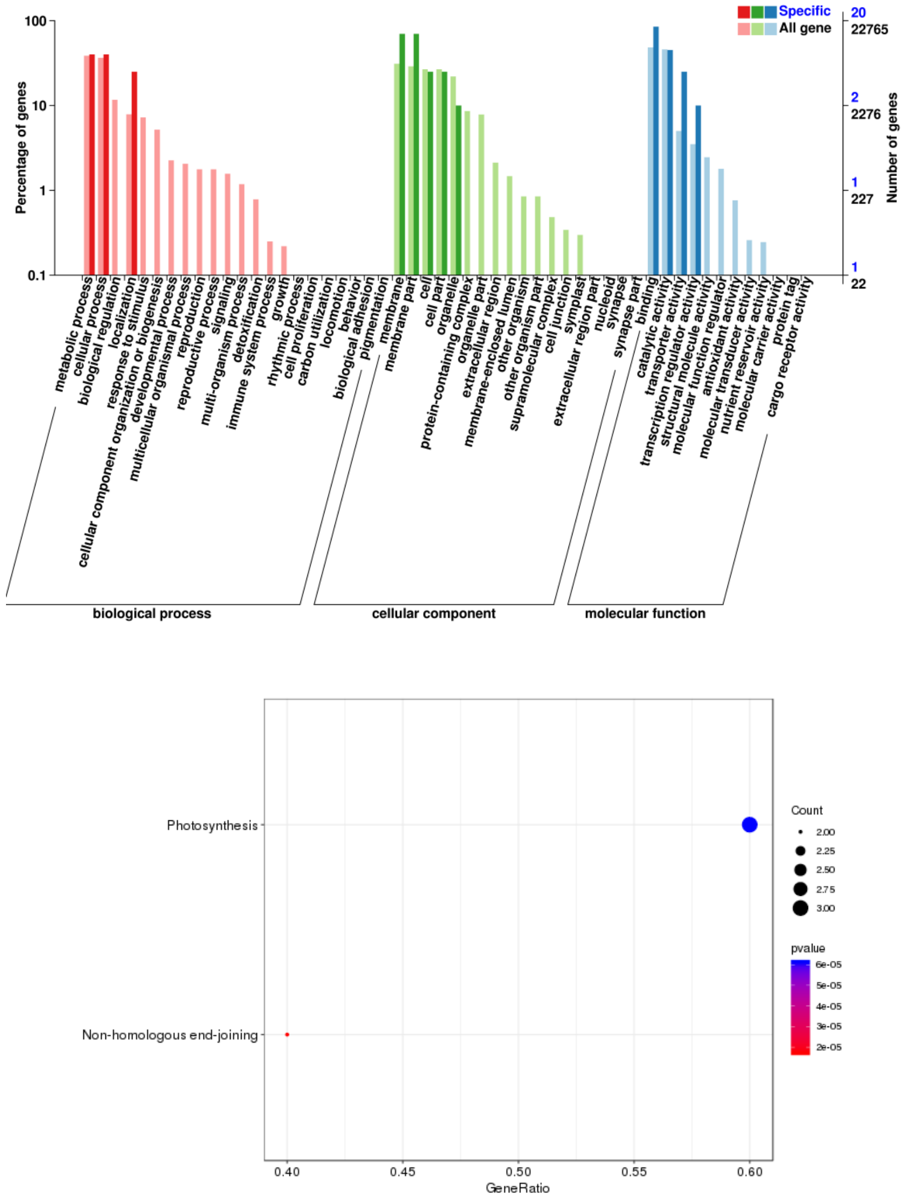

Supplementary Figure 14. Species-specific orthogroups of DB (GO and KEGG annotation)

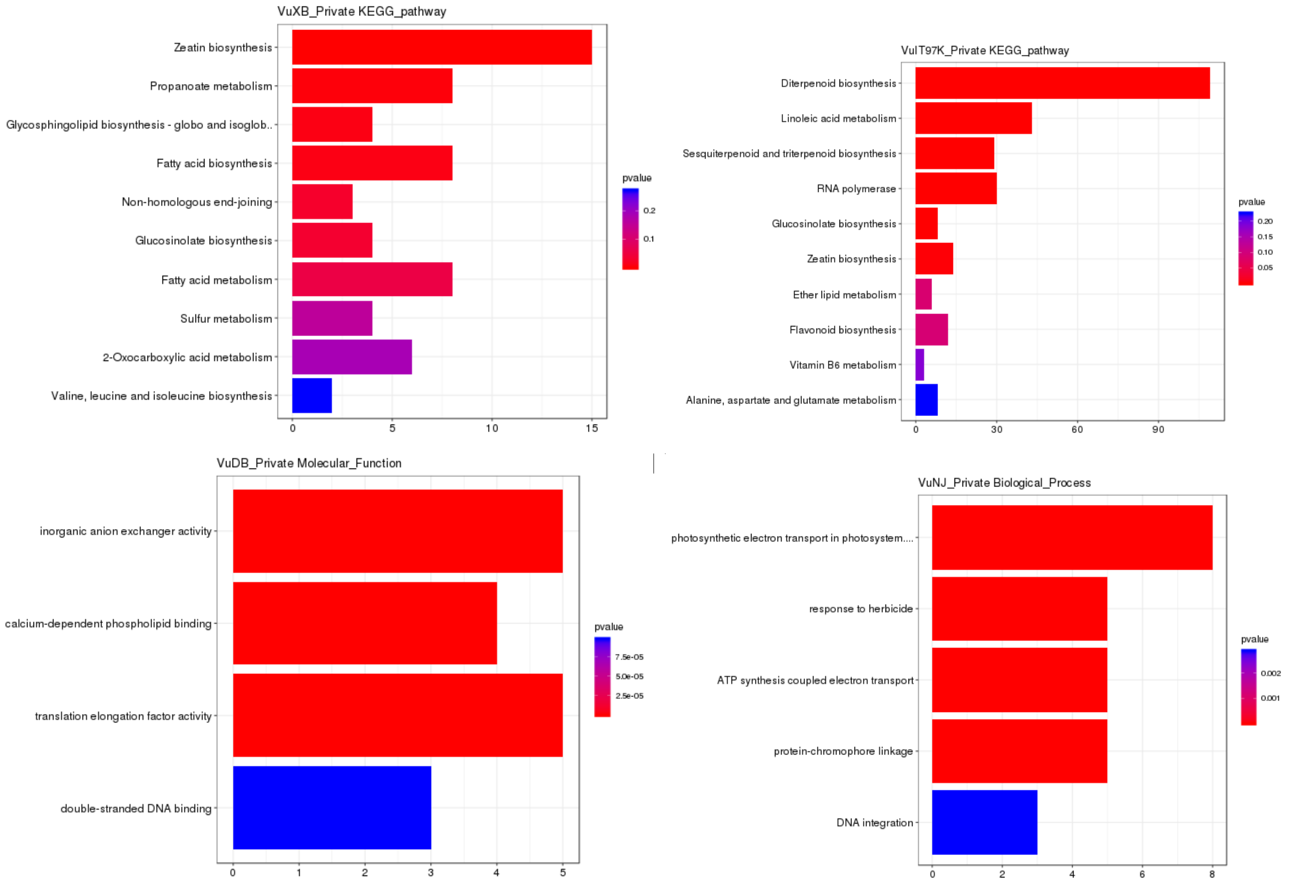

Supplementary Figure 15. Private Genome KEGG Enrichment of Four Varieties (Up left—Xiabao II, Up right—IT97K-499-35, down left—DB, down right—NJ)

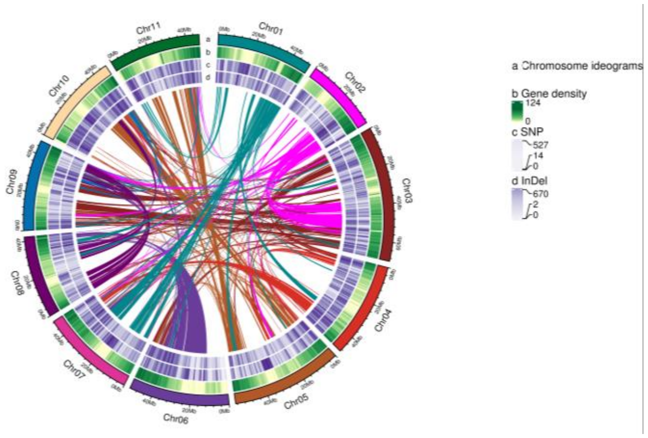

Supplementary Figure 16. Circle plot of SNP and InDel variant site density in the pan-genome. a. Chromatology number. b. Gene density distribution. C. SNP density distribution. D. Indel density distribution. The inner circle represents the common linearity of the NJ genome.

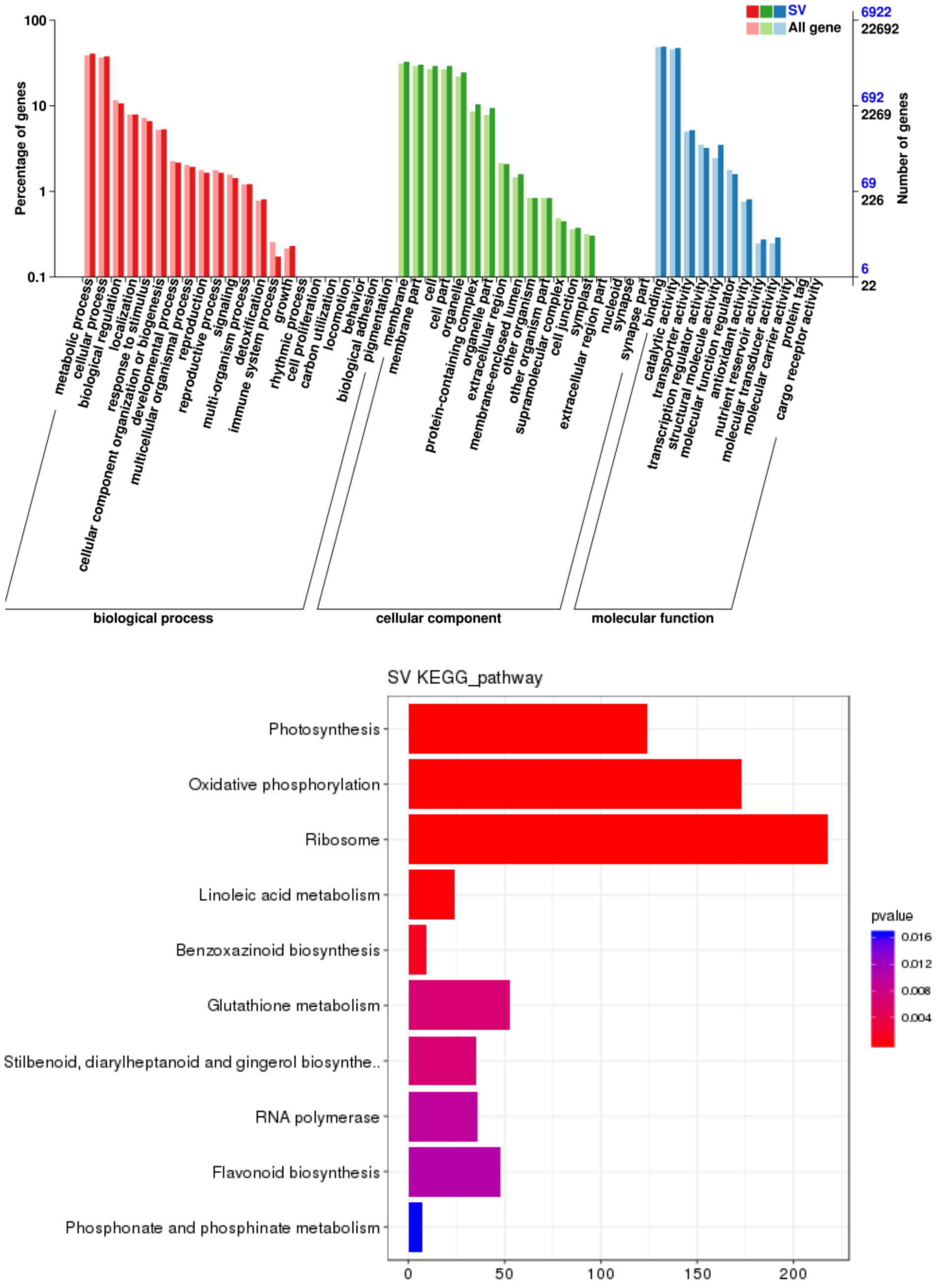

Supplementary Figure 17. GO and KEGG enrichment of genes affected by SV variants

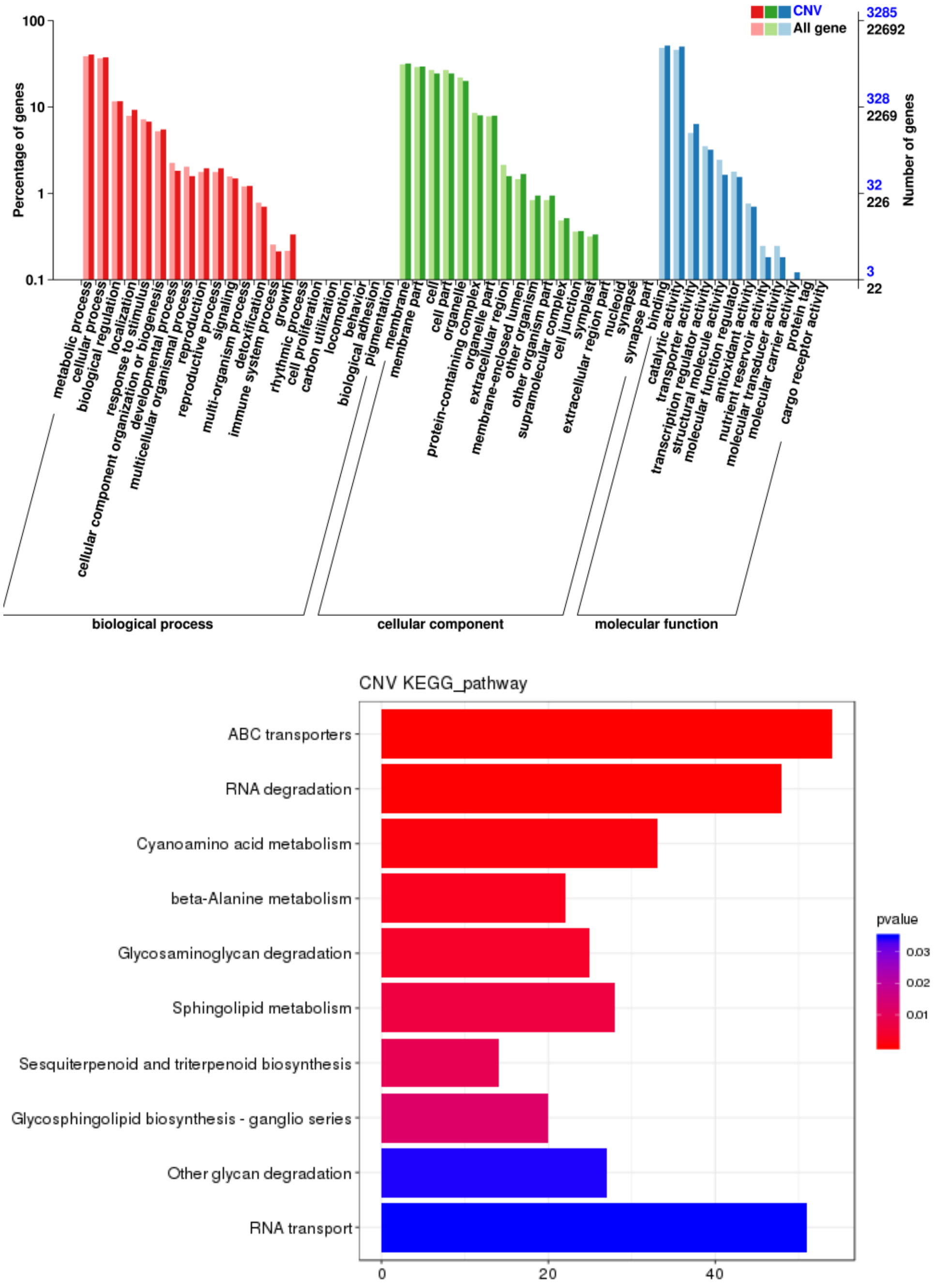

Supplementary Figure 18. GO and KEGG enrichment of genes affected by CNV variants

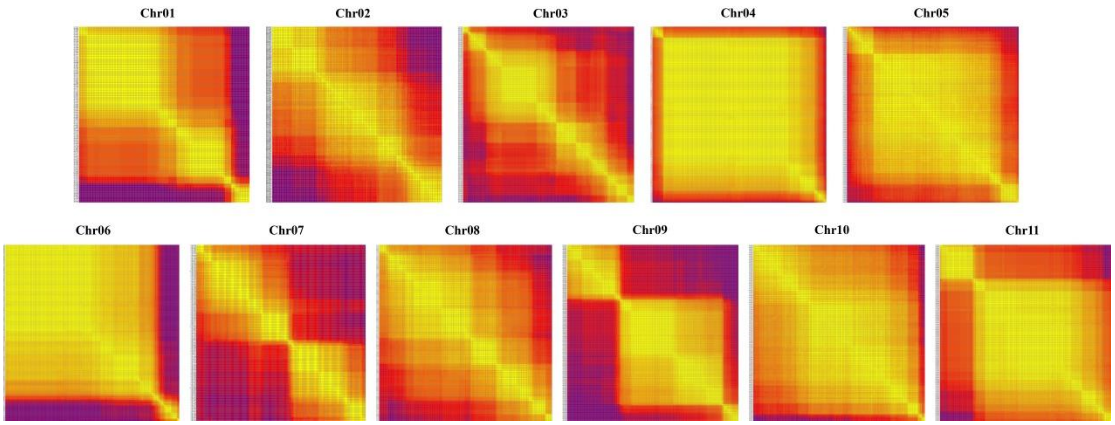

Supplementary Figure 19. Heatmap for high-density genetic map assessment

2.2 Supplementary Tables

Supplementary Table 1 HiFi Genome Assembly Evaluation

Genomic Evaulation		NJ	DB
Data Return Ratio	Total reads	230772782	220752200
	Mapped reads	228840934	218458170
	Mapped %	99.16	98.96
	Properly mapped reads	227664082	2171607 0
	Properly mapped %	98.65	98.37
CEGMA Evaluation	Complete BUSCOs(C)	1602 (99.26%)	1597 (98.95%)
	Complete and single-copy BUSCOs(S)	1554 (96.28%)	1551 (96.10%)
	Complete and duplicated BUSCOs(D)	48 (2.97%)	46 (2.85%)
	Fragmented BUSCOs(F)	3 (0.19%)	4 (0.25%)
	Missing BUSCOs(M)	9 (0.56%)	13 (0.81%)
	Total Lineage BUSCOs	1614	1614
BUSCO Evaluation	Number of 458 CEG* present in assembly	452	452
	%of 458 CEGs present in assemblies	98.69%	98.69%
	Number of 248 highly conserved CEGs present	227	229
	% of 248 highly conserved CEGs present	91.53%	92.34%

Supplementary Table 2 Hi-C sequencing data

Genome feature	NJ	DB
Read Sum	351,890,227	709,464,025
Clean Data	105,320,008,060	212,233,466,456
Longest Scaffold (bp)	69560973	69522628
Longest Scaffold (bp)	53847691	51375582
Q20%	98.21	97.49
Q30%	94.47	92.62

Total Read Pairs	315,890,227	709,464,025
Mapped Reads	642,435,885	1,264,992,049
Umnique Mapped Reads Pairs	298,094,430	577,155,351

Supplementary Table 3 Transposable elements statistics (NJ)

	Type	Number	Length	Rate(%)
**ClassI**	Retroelement	293033	173337341	31.5
	DIRS	5	153	0
	LINE	25970	6312388	1.15
	LTR/Caulimovirus	149	278690	0.05
	LTR/Copia	52593	34975317	6.36
	LTR/ERV	2951	261814	0.05
	LTR/Gypsy	96323	85824592	15.6
	LTR/Ngaro	790	63449	0.01
	LTR/Pao	76	5027	0
	LTR/Unknown	111864	45289357	8.23
	SINE	2312	326554	0.06
**ClassII**	DNA transposon	198577	53361941	9.7
	Academ	1	72	0
	CACTA	18947	4619212	0.84
	Crypton	318	12390	0
	Dada	2125	100618	0.02
	Ginger	730	32008	0.01
	Helitron	3306	2302323	0.42
	IS3EU	449	24033	0
	Kolobok	3665	275763	0.05
	Maverick	240	27829	0.01
	Merlin	1424	62525	0.01
	Mutator	3334	1554943	0.28
	P	1285	78106	0.01
	PIF-Harbinger	4229	233496	0.04
	PiggyBac	917	41470	0.01
	Tc1-Mariner	724	37626	0.01
	Unknown	136609	39702330	7.21
	Zator	1	61	0
	Zisupton	93	3297	0
	hAT	20180	4253839	0.77
Unknown		18	1267	0
Total		491628	226700549	41.19

Supplementary Table 4 Transposable elements statistics (DB)

	Type	Number	Length	Rate(%)
ClassI	Retroelement	293952	174375278	30.91
	DIRS	4	159	0
	LINE	31232	7833942	1.39
	LTR/Caulimovirus	583	539615	0.1
	LTR/Copia	47590	33347318	5.91
	LTR/ERV	4322	506259	0.09
	LTR/Gypsy	97810	88047728	15.61
	LTR/Ngaro	1042	132307	0.02
	LTR/Pao	352	66838	0.01
	LTR/Unknown	109663	43710285	7.75
	SINE	1354	190827	0.03
ClassII	DNA transposon	218012	59198335	10.49
	Academ	2	145	0
	CACTA	17662	4411901	0.78
	Crypton	301	13535	0
	Dada	1985	89836	0.02
	Ginger	739	32056	0.01
	Helitron	3012	2176091	0.39
	IS3EU	469	28852	0.01
	Kolobok	3197	210255	0.04
	Maverick	316	37199	0.01
	Merlin	1313	57550	0.01
	Mutator	4460	2497895	0.44
	P	1219	73263	0.01
	PIF-Harbinger	3979	213714	0.04
	PiggyBac	890	38032	0.01
	Tc1-Mariner	631	31497	0.01
	Unknown	157890	45120994	8
	Zator	1	61	0
	Zisupton	102	3732	0
	hAT	19844	4161727	0.74
	Unknown	21	1345	0
	Total	511985	233574958	41.41

Supplementary Table 5 Tandem Duplications Prediction Statistics

Type	NJ			DB		
	Number	Length	Rate(%)	Number	Length	Rate(%)
microsatellite (1-9 bp units)	227274	7224719	1.31	225080	8648523	1.53
minisatellite (10-99 bp units)	121038	11357166	2.06	136888	13086467	2.32
satellite (>=100 bp units)	11735	41940026	7.62	13605	51857323	9.19
Total	360047	60521911	11.00	375573	73592313	13.05

Supplementary Table 6 Motif annotation statistics

Accession	motif	domain
DB	1283	30281
NJ	1279	30228

Supplementary Table 7 Transcriptome data evaluation

Accession	Obtained Reads	OBtained Base(bp)	Q20(%)	Q30(%)	GC(%)
NJ	39,213,677	11,700,554,188	98.37	94.97	44.85
DB	34,554,208	10,319,636,688	98.30	94.74	43.91

Supplementary Table 8 BUSCO assesses the genome

Accession	Complete BUSCOs	Complete and single-copy BUSCOs	Complete and duplicated BUSCOs	Fragmented BUSCOs	Missing BUSCOs	Total Lineage BUSCOs
DB	1574 (97.52%)	1524 (94.42%)	50 (3.10%)	29 (1.80%)	11 (0.68%)	1614
NJ	1586 (98.27%)	1540 (95.42%)	46 (2.85%)	19 (1.18%)	9 (0.56%)	1614

Supplementary Table 9 Gene function annotation statistics

Annotation Database	NJ		DB	
	Annotated Number	Annotated Ratio	Annotated Number	Annotated Ratio
GO Annotation	22692	79.99	22765	80.09
KEGG Annotation	20109	70.88	20166	70.94
KOG Annotation	14413	50.80	14475	50.92
Pfam Annotation	23522	82.91	23590	82.99
Swissprot Annotation	22320	78.67	22394	78.78
TrEMBL Annotation	27482	96.87	27527	96.84
eggNOG Annotation	22553	79.50	22599	79.50
nr Annotation	26972	95.07	27033	95.10
All Annotated	27491	96.90	27538	96.88

Supplementary Table 10 GO enrichment of positive selection gene families in genome (P <0.05)

ID	Description	Gene Ratio	enrich factor	p-value
		NJ		
GO:0051173	positive regulation of nitrogen compound metabolic process	2/44	7.81	0.027
GO:0010604	positive regulation of macromolecule metabolic process	2/44	7.42	0.030
GO:0031325	positive regulation of cellular metabolic process	2/44	7.42	0.030
GO:0009893	positive regulation of metabolic process	2/44	6.73	0.035
GO:0048518	positive regulation of biological process	3/44	4	0.039
GO:0006260	DNA replication	2/44	6.25	0.041
GO:0000272	polysaccharide catabolic process	1/44	21.88	0.045
GO:0006379	mRNA cleavage	1/44	21.88	0.045
GO:0008214	protein dealkylation	1/44	21.88	0.045
GO:0016577	histone demethylation	1/44	21.88	0.045
GO:0022618	ribonucleoprotein complex assembly	1/44	21.88	0.045
GO:0031401	positive regulation of protein modification process	1/44	21.88	0.045
GO:0043631	RNA polyadenylation	1/44	21.88	0.045
GO:0070076	histone lysine demethylation	1/44	21.88	0.045
GO:0071826	ribonucleoprotein complex subunit organization	1/44	21.88	0.045
GO:1902903	regulation of supramolecular fiber organization	1/44	21.88	0.045
GO:0005975	carbohydrate metabolic process	5/44	2.49	0.049
GO:0006012	galactose metabolic process	1/44	19.89	0.049
GO:0006270	DNA replication initiation	1/44	19.89	0.049
GO:0006378	mRNA polyadenylation	1/44	19.89	0.049
GO:0010639	negative regulation of organelle organization	1/44	19.89	0.049
GO:0045861	negative regulation of proteolysis	1/44	19.89	0.049
		DB		
GO:0006261	DNA-dependent DNA replication	2/64	13.14	0.010
GO:0008033	tRNA processing	2/64	9.16	0.020
GO:0006281	DNA repair	3/64	4.49	0.029
GO:0022402	cell cycle process	2/64	5.93	0.045

Supplementary Table 11 KEGG enrichment of positive selection gene families in genome ($P < 0.05$)

ID	Description	Gene Ratio	Enrich factor	p-value
		NJ		
ko03015	mRNA surveillance pathway	4/48	5.46	0.006
ko00565	Ether lipid metabolism	2/48	10.14	0.016
ko00513	Various types of N-glycan biosynthesis	2/48	7.34	0.030
ko00510	N-Glycan biosynthesis	2/48	5.68	0.048
		DB		
ko03430	Mismatch repair	4/66	8.62	0.001
ko03030	DNA replication	3/66	5.75	0.015
ko03040	Spliceosome	6/66	2.71	0.023
ko03420	Nucleotide excision repair	3/66	4.66	0.027
ko00790	Folate biosynthesis	2/66	5.64	0.049

Supplementary Table 12 Pan-genome composition statistics

Species	Core	Dispensable	Private	Total
Xiabao II	22592	3801	9259	35652
IT97K-499-35	21861	3816	1751	27428
DB	21749	6356	28	28133
NJ	21728	6348	53	28129

Supplementary Table 13 Pan-genome composition statistics

Species	Gene	Intergenic	TotalNumber	TotalLength
Xiabao II	155245	718964	874209	874209
IT97K-499-35	432899	1188202	1621101	1621101
DB	294414	1244596	1539010	1539010

Supplementary Table 14 SNP and InDel structural annotation

	Species	UTR'5	UTR'3	Downstream	Upstream	Intronic	Exonic	Intergenic	Splicing
SNP	Xiabao II	3020	4755	30745	36293	2404	56699	21271	718964
	IT97K-499-35	9031	13662	87537	102394	6063	156278	57848	1188202
	DB	5856	9880	58594	70455	4130	106333	39105	1244596
InDel	Xiabao II	1766	2912	18934	22401	1605	27661	4397	335322
	IT97K-499-35	3630	5328	37452	45102	2897	55174	3513	364342
	DB	2139	3312	22709	28575	1836	34626	2013	238214

Supplementary Table 15 Gene exon variation Statistics

	Functional variation	Xiabao II	IT97K-499-35	DB
SNP	nonsynonymous SNV	11520	29141	19524
	startloss	25	45	34
	stopgain	243	290	186
	stoploss	35	63	41

	synonymous SNV	19320	28309	19320
InDel	frameshift deletion	518	1397	486
	frameshift insertion	3076	658	558
	nonframeshift deletion	271	641	431
	nonframeshift insertion	256	597	410
	startloss	2	10	13
	stopgain	239	175	93
	stoploss	11	35	22

Supplementary Table 16 Number and length statistics of PAVs, SVs, and CNVs in the pan-genome

Category		Xiabao II	DB	IT97K-499-35
PAV	Presence Number	3,461	2,367	3,688
	Presence Length	2,809,457	1,263,236	2,058,013
	Absence Number	2,129	2,360	3,710
	Absence Length	1,119,481	903,351	1,986,830
	Total Number	5,590	4,727	7,398
	Total Length	3,928,938	2,166,587	4,044,843
SV	TRANS Number	1,974	509	891
	INV Number	113	35	59
	DUP Number	5,892	2,285	4,032
	Total Number	7,979	2,829	4,982
CNV	CPG	22,334	537	767
	CPL	402	515	753
	Total	22,736	1,052	1,520

Supplementary Table 18 SLAF-tags on each chromosome

ChrID	SLAF Number	Polymorphic SLAF
Chr01	25,545	5,354
Chr02	19,529	2,203
Chr03	34,536	2,965
Chr04	26,396	3,926
Chr05	30,058	3,087
Chr06	24,481	3,313
Chr07	25,885	1,325
Chr08	23,362	1,448
Chr09	23,706	1,431
Chr10	26,004	2,894
Chr11	23,970	1,945
Total	283,472	29,891

Supplementary Table 19 Basic information of linkage map

LGID	Marker Number	Total Distance	Average Distance	Gaps ≦5	Max Gap
Chr01	269	75.14	0.28	98.51%	9.73
Chr02	223	141.28	0.64	100%	3.92
Chr03	288	123.24	0.43	99.30%	6.23
Chr04	773	91.58	0.12	99.87%	6.44
Chr05	333	135.93	0.41	99.40%	5.16
Chr06	775	121.55	0.16	100%	3.81
Chr07	197	122.01	0.62	98.98%	6.44
Chr08	451	112.36	0.25	100%	4.21
Chr09	318	110.88	0.35	99.05%	6.43
Chr10	329	127.73	0.39	99.39%	7.13
Chr11	301	91.41	0.3	99.67%	6.33
Total	4,257	1,253.11	0.3	99.47%	9.73

Supplementary Table 20 Statistical of SNP marker information on the map

LG ID	SNP Number	Trv	Tri	Trv/Tri
Chr01	462	164	298	0.55
Chr02	390	142	248	0.57
Chr03	451	133	318	0.42
Chr04	1,674	492	1,182	0.42
Chr05	595	148	447	0.33
Chr06	1,294	368	926	0.40
Chr07	309	125	184	0.68
Chr08	612	196	416	0.47
Chr09	467	170	297	0.57
Chr10	730	234	496	0.47
Chr11	555	159	396	0.40
Total	7,539	2,331	5,208	0.45
